# The *Vibrio alginolyticus* T3SS effectors, Val1686 and Val1680, induce cell rounding, apoptosis and lysis of fish epithelial cells

**DOI:** 10.1080/21505594.2017.1414134

**Published:** 2018-02-27

**Authors:** Zhe Zhao, Jinxin Liu, Yiqin Deng, Wen Huang, Chunhua Ren, Douglas R. Call, Chaoqun Hu

**Affiliations:** a Institute of Marine Biology, College of Oceanography, Hohai University, Nanjing, Jiangsu, PR China; b Key Laboratory of Marine Bio-resources Sustainable Utilization, Key Laboratory of Applied Marine Biology of Guangdong Province, South China Sea Institute of Oceanology, Chinese Academy of Sciences, Guangzhou, Guangdong, PR China; c Paul G. Allen School for Global Animal Health, Washington State University, Pullman, WA, U.S; d Key Laboratory of South China Sea Fishery Resources Exploitation & Utilization, Ministry of Agriculture, South China Sea Fisheries Research Institute, Chinese Academy of Fishery Sciences, Guangzhou, Guangdong, PR China

**Keywords:** *Vibrio alginolyticus*, T3SS, effector protein, apoptosis, fish disease

## Abstract

*Vibrio alginolyticus* is a Gram-negative bacterium that is an opportunistic pathogen of both marine animals and people. Its pathogenesis likely involves type III secretion system (T3SS) mediated induction of rapid apoptosis, cell rounding and osmotic lysis of infected eukaryotic cells. Herein, we report that effector proteins, Val1686 and Val1680 from *V. alginolyticus*, were responsible for T3SS-mediated death of fish cells. Val1686 is a Fic-domain containing protein that not only contributed to cell rounding by inhibiting Rho guanosine triphosphatases (GTPases), but was requisite for the induction of apoptosis because the deletion mutant (Δ*val1686*) was severely weakened in its ability to induce cell rounding and apoptosis in fish cells. In addition, Val1686 alone was sufficient to induce cell rounding and apoptosis as evidenced by the transfection of Val1686 into fish cells. Importantly, the Fic-domain essential for cell rounding activity was equally important to activation of apoptosis of fish cells, indicating that apoptosis is a downstream event of Val1686-dependent GTPase inhibition. *V. alginolyticus* infection likely activates JNK and ERK pathways with sequential activation of caspases (caspase-8/-10, -9 and -3) and subsequent apoptosis. Val1680 contributed to T3SS-dependent lysis of fish cells in *V. alginolyticus*, but did not induce autophagy as has been reported for its homologue (VopQ) in *V. parahaemolyticus*. Together, Val1686 and Val1680 work together to induce apoptosis, cell rounding and cell lysis of *V. alginolyticus*-infected fish cells. These findings provide new insights into the mechanism of cell death caused by T3SS of *V. alginolyticus*.

## Introduction

The type III secretion system (T3SS) is a well-characterized needle-like apparatus that is found on the surface of many Gram-negative bacteria, and it plays a critical role in pathogenesis [1]. T3SSs are composed of a basal body that spans the inner and outer bacterial membranes, a needle-like complex that extends from the bacterial surface, and a distal needle tip complex that forms a channel into the target cell membrane [2,3]. Effector proteins are translocated by this apparatus where they modulate cytoskeletal rearrangement or perturb cellular signaling pathways that are involved with host cell death and innate immune responses [4,5]. Although structural proteins are highly conserved among different T3SS apparatus, the effectors are most often distinct and exhibit unique functions [6]. Characterization of the structure and function of various effector proteins is important to understand the pathogenesis of these important pathogens.

T3SS was first discovered in *Yersinia* [7]. Since then presumptive orthologues have been identified in other bacterial pathogens, including species of *Escherichia, Pseudomonas, Shigella, Salmonella, Xanthomonas, Bordetella, Burkholderia* and *Chlamydia* [5]. For *Vibrio* spp., two distinct T3SSs were originally idenitified in the genome of *Vibrio parahaemolyticus* (serotype O3:K6, strain RIMD2210633); each of the two *V. parahaemolyticus* chromosomes encodes a distinct T3SS, designated T3SS1 and T3SS2^8^. T3SS1 has been found in all strains of *V. parahaemolyticus* examined [9] and is necessary for *in vitro* cytotoxicity including the induction of autophagy, cell rounding and cell lysis [10,11]. T3SS2 has been found exclusively in clinical isolates (Kanagawa Phenomenon-positive) of *V. parahaemolyticus* [8,9], and it is required for induction of diarrhea and enteritis in rabbit and piglet [9,12,13]. To date, four T3SS1 effectors (VopS, VopQ, VopR and VPA0450) have been identified. VopS is required for T3SS1-induced actin cytoskeleton collapse and cell rounding, which is a phenotype that is induced by modifying the Rho family GTPases through AMPylation [14,15]. Rho GTPases belong to the Ras superfamily of monomeric GTP-binding proteins and are best known for their prominent roles in regulating actin and microtubule cytoskeletal dynamics [16,17]. VopQ (Vp1680) was responsible for induction of rapid autophagy in HeLa cells. The mechanism involves an interaction with the Vo domain of the conserved V-type H^+^-ATPase (V-ATPase) that forms a gated channel in lysosomal membranes [10,18,19]. VopR (VP1683) also contributes to cell rounding [15,20] while VPA0450 disrupt plasma membrane integrity and facilitates lysis of host cells [21,22]. Less is known about T3SS orthologues from other phylogenetically-related *Vibrio* species, including *Vibrio alginolyticus*.

*V. alginolyticus* is closely related to *V. parahaemolyticus* [23]. *V. alginolyticus* is a common marine organism that can cause opportunistic infections in aquatic animals and people [24,25]. In south coastal areas of China, *V. alginolyticu*s is the most prevalent *Vibrio* species and it is responsible for large losses to the marine aquaculture industry [26,27]. Several virulence factors, including the iron uptake system, haemolysin and extracellular proteases, likely play a role in its pathogenesis [28-31]. Recently, a putative T3SS “island” was identified in *V. alginolyticus* (ZJO, one disease-causing strain), and this island was similar in synteny and predicted protein composition to T3SS1 characterized in *V. parahaemolyticus [*32]. Further studies revealed that apoptosis, cell rounding and osmotic lysis are involved in *V. alginolyticus* T3SS-induced cell death [32]. The death process in fish cells was different from that caused by *V. parahaemolyticus* in mammalian cells as the latter induces autophagy rather than apoptosis, although the mechanisms of cell lysis appears similar [10,11]. Comparative genome analysis of the T3SS gene cluster from *V. alginolyticus* suggest that Val1686 and Val1680 are orthologues of VopS and VopQ in *V. parahaemolyticus*, but their function has not been characterized. Herein, we investigated the functional activity of Val1686 and Val1680 in *V. alginolyticus* by using a fish-cell infection model to further explore the fundamental mechanism of its pathogenic mechanisms.

## Materials and methods

### Bacterial strains, plasmids and growth conditions

The bacterial strains and plasmids used in this study are listed in Table S1. All *V. alginolyticus* strains were derived from the wild-type strain, ZJO. *V. alginolyticus* was routinely grown in Trypticase Soy Broth (TSB; Difco) with shaking (200 rpm) or on TSB agar plates (TSA) at 30°C. T3SS secretion was induced by culturing bacteria in TSB supplemented with 10 mM MgCl_2_ and 10 mM sodium oxalate [10]. *Escherichia coli* S17 λ*pir* was used in gene deletion experiments and was cultured in Luria-Bertani (LB; Difco) medium. Expression vector pMMB207 was used for complementation experiments and suicide plasmid pDM4 was used to generate gene knockouts. Expression vectors (pEGFP-N3 and pcDNA3.1) were used to express genes of interest in fish cells. Unless otherwise indicated, antibiotics were added to media at the following concentrations: ampicillin (100 μg/mL), kanamycin (50 μg/mL), or chloramphenicol (34 μg/mL).

### Construction of deletion mutants and complementation strains

All deletion mutants were made by allelic exchange following a method described previously [33]. Primer pairs used for plasmid construction in this study are detailed in Table S2. Deletion cassettes for chromosomal in-frame deletions were generated using the splice-overlap-extension (SOE) method, which joins two 400–600 bp PCR fragments corresponding to genomic sequences flanking *val1686* or *val1680*. The deletion cassettes were then cloned into a suicide plasmid (pDM4) by using standard cloning procedures followed by DNA sequencing (Table S2). The resulting constructs were individually electroporated into *E. coli* S17-1 λpir, after which the constructs were introduced by conjugation into *V. alginolyticus* strain ZJO. Mutant strains were selected on TSA plates containing ampicillin and chloramphenicol followed by a 10 % sucrose selection process. Gene deletion was confirmed by PCR using primers located inside of the deleted sequence (Table S2).

For complementation experiments, the complete *val1686* and *val1680*, or truncated *val1686* (1-90 deletion) incorporating a C-terminal histidine tag by PCR, were cloned into an expression vector pMMB207 by using standard cloning procedures. For site-directed mutagenesis, primers (Table S2) were designed by using NEBaseChanger (http://nebasechanger.neb.com/) and were then used to generate point mutation and small deletion plasmids (Table S1) with a Q5 Site-Directed Mutagenesis Kit (New England Biolabs) following the manufacturer's protocol. These constructs were fully sequenced to check their inserts and then introduced by conjugation into the appropriate mutant strains.

### Cell lines and infection

Fathead minnow (FHM) epithelial cells were maintained in M199 medium supplemented with 10% (v/v) fetal bovine serum (FBS, Gibco) at 28°C. Overnight *V. alginolyticus* cultures were pelleted by centrifugation (10,000 × *g*) for 2 min at 4°C. Pellets were resuspended in serum-free TC199 medium, and bacterial suspensions were added to the FHM monolayer at a multiplicity of infection (MOI) of 10. For infection with complementation strains, overnight culture was diluted 1:100 into fresh TSB with chloramphenicol and incubated with shaking until OD_600_ reached ∼0.6. Isopropyl β-D-1-thiogalactopyranoside (IPTG; 0.5 mM) was then added to induce protein expression. After 3 h the induced bacteria were added to the FHM monolayer supplemented with 5 μg/mL of chloramphenicol to ensure plasmid retention [34] and 0.1 mM of IPTG.

### GTPase pulldown assay

FHM cells uninfected (Ctrl) or infected with *V. alginolyticus* ZJO and ZJOΔ*val1686* (Δ*val1686*) for the indicated time points were assayed for GTPase activation using commercially available Rho and Cdc42 Activation Assay Kits (Cell Biolabs, Inc.) according to the manufacturer's instructions. Briefly, FHM cells were seeded onto 100 mm dishes. Once cells were cultured to approximately 80–90% confluency, they were infected with *V. alginolyticus* as described above. Cells monolayers were washed twice with ice-cold PBS and collected in lysis buffer (25 mM HEPES [pH 7.5], 150 mM NaCl, 1% NP-40, 10 mM MgCl_2_, 1 mM EDTA, 1% glycerol) by scraping with a cell scraper. All samples were then lysed by incubating in ice-water with agitation for 20 min. The lysates were cleared of insoluble debris by centrifugation for 10 min (14,000 × *g* at 4°C). For GTPase activation, cleared lysates were inoculated with a final concentration of 1 mM EDTA and 100 μM GTPγS and incubated at 30°C for 30 min with agitation. Activated Rho and Cdc42 GTPases were affinity purified by using Rhotekin-RBD and PAK-1agarose beads, respectively. The beads were washed three times with lysis buffer to remove inactive GTPases and other cellular proteins followed by the addition of SDS-PAGE sample buffer. Total cellular GTPases were also prepared in a similar manner without the addition of Rhotekin-RBD and PAK-1 agarase beads. All samples were separated on SDS-PAGE and analyzed by using western blots (see below). The primary antibodies were monoclonal antibodies to Rho and Cdc42 provided with the kit (1:500 dilutions).

### Fluorescence microscopy

To fluorescently stain the actin cytoskeleton and nuclear DNA of infected cells, FHM cells were seeded onto glass coverslips in 6-well plates and grown to 80–90% confluence prior to infection. All infections were performed as described above, and at the indicated time points, cells were fixed and stained with rhodamine phalloidin (Molecular Probes) and Hoechst33258 (Thermo Scientific) as described previously [35]. Slides were mounted in ProLong Gold Antifade Reagent (Molecular Probes). The fluorescence signal was detected by using an inverted fluorescence microscope (Leica).

### Cell transfection

The plasmids used for transfection experiments (Table S1) were constructed by inserting the complete *val1686* or truncated *val1686* (1–90 deletion) sequence into eukaryotic expression vectors (pEGFP-N3 and pcDNA3.1) using standard cloning procedures. A point mutation plasmid was generated by using a Q5 Site-Directed Mutagenesis Kit (New England Biolabs) as described above. Each construct (0.5 µg of DNA/well for 24-well plate and 2 µg of DNA/well for 6-well plate) was individually transfected into FHM cells using Lipofectamine 2000 Transfection Reagent (Invitrogen) according to the manufacturer's instructions. FHM cells were plated onto glass coverslips in a 6-well plate with 60–70% confluence one day before transfection. Cells were prepared for a caspase activity assay (see below) by seeding FHM cells into a 24-well plate with 90% confluence one day before transfection. Eight hours after transfection, the cells on glass coverslips were fixed and stained with Hoechst 33258 (Thermo Scientific) and then the slides were examined by fluorescence microscopy as described above, while the cells in a 24-well plate were collected for measuring caspase activity.

### TUNEL assay

DNA fragmentation was detected by using the terminal dUTP nick-end labelling (TUNEL) method and a commercial kit (*In situ* Cell Death Detection kit, TMR red; Roche) following the manufacturer's protocol. FHM cells were infected with strains of *V. alginolyticus* as described, or left untreated as the negative control. The assay was performed as described previously [32].

### Caspase activity assay

The activities of several caspases (caspase-3, -8, -9 and -10) were detected using commercially available caspase assay kits (BioVision). FHM cells were left uninfected, or infected with *V. alginolyticus* strains for 2 h as described, and were then lysed on ice for 10 min with the cell lysis buffer provided in the kit. All assays were done as described previously [32].

### *In vitro* secretion assay

To induce the *V. alginolyticus* T3SS secretion, overnight cultures were diluted 1:100 into the inducing TSB medium as described (with chloramphenicol) and were cultured at 30°C with shaking. When OD_600_ reached ∼0.6, IPTG (0.5 mM) was added to induce protein expression and then continued to grow for 5 h. Bacterial cells were pelleted by centrifugation (3200 × *g*, 10 min), and were then re-suspended in Laemmli sample buffer (Bio-rad). The supernatant was collected and filtered through a low-protein-binding filter with a 0.2-µm pore size (Acrodisc syringe filter; Pall Life Sciences). Trichloroacetic acid (TCA) precipitation was carried out as described previously [36,37] with a minor modification. Briefly, TCA was added to a final volume of 20% and incubated in ice-water for 2 h. After centrifugation at 22,000 × *g* for 30 min at 4°C, the pellets were resuspended in pre-chilled acetone and washed twice. The final protein pellet was dried in air and re-suspended in Laemmli sample buffer (Bio-Rad). All samples were boiled for 8 min and analyzed by western blots (see below). The primary antibodies were monoclonal antibody to His tag (1:2000 dilution; Abcam) or polyclonal antibody to DnaK (1:1000 dilution) [38].

### Western blot analysis

An equal volume of 2X Laemmli sample buffer (Bio-Rad) was added into each sample and all samples were boiled at 100°C for 5 min. Equal volume of prepared samples were loaded into AnykD Tris-glycine precast gels (Bio-Rad) for protein separation per manufacturer's protocol. After electrophoresis proteins were transferred to a PVDF membrane (Roche) and the membrane was blocked with 5% skimmed milk in Tris-buffered saline containing 0.05% (v/v) Tween 20 (TBST; Sangon Biotech). After 1 h blocking, the membrane was respectively probed with primary antibodies as indicated for 2 h at room temperature. Secondary antibody (HRP-linked goat anti-mouse antibody or goat anti-rabbit antibody, Cell Signaling Technology) was diluted 1:5000 in TBST with 1% milk for 1 h at room temperature. Protein signals were detected with a chemiluminescent reagent (ECL; SuperSignal West Pico Chemiluminescent substrate; Thermo Scientific) followed by exposure of membranes to autoradiograph film (Kodak X-Omat LS film).

### Inhibitor assay

U0126-EtOH, SB203580 and SP600125, which inhibit pathways involving ERK, p38 and JNK MAPK, respectively, were purchased from Selleck. Next-generation caspase inhibitors (Q-IETD-OPh and Q-LEHD-OPh) and a control inhibitor Q-VE-OPh were purchased from BioVision. These reagents were prepared as stock solutions by dissolving in dimethyl sulfoxide (DMSO). All inhibitors were added to cell monolayers grown in 24-well plates for 1 h before infection with *V. alginolyticus*. To rule out effect of DMSO on apoptosis, the same volume of DMSO was respectively added to the uninfected and ZJO-infected cells as negative and positive controls. The final concentration of each inhibitor used here showed no cytotoxicity to FHM cells, as tested by MTT cell viability assay. To evaluate the effect of inhibitors after 2 h of infection, cultures were subjected to TUNNEL and caspase activity assays as described above.

### Measurement of lactate dehydrogenase (LDH) release

FHM cells were seeded into 96-well plates and incubated overnight to 90% confluence. Prior to infection, growth media was replaced with 110 μL (per well) of serum-free TC199 medium, and cells were infected with different strains of *V. alginolyticus* over a 5-h time course. At the indicated time point, 96-well plates were centrifuged at 3200 × *g* for 2 min, and a 100 µL aliquot of the supernatant was removed for measuring LDH release by using the Cytotoxicity Detection kit^PLUS^ per manufacturer's instructions (Roche). Background LDH release was measured from TC199 medium only. Maximum LDH release was obtained by total cell lysis using the Lysis Buffer provided in the kit. Absorbance values from each well were measured at 492 nm by using a microplate spectrophotometer (Thermo Labsystems Ascent). Results are expressed as a percentage of total cell lysis after substracting the absorbance value in background control.

### Statistical analysis

A Kruskal-Wallis test was used to assess phenotypic variables (α = 0.05). Post-hoc multiple comparisons (versus the control group) were conducted by using a Tukey-Kramer test and the analyses were conducted by using R (version 3.4.1).

## Results

**Figure 1.**
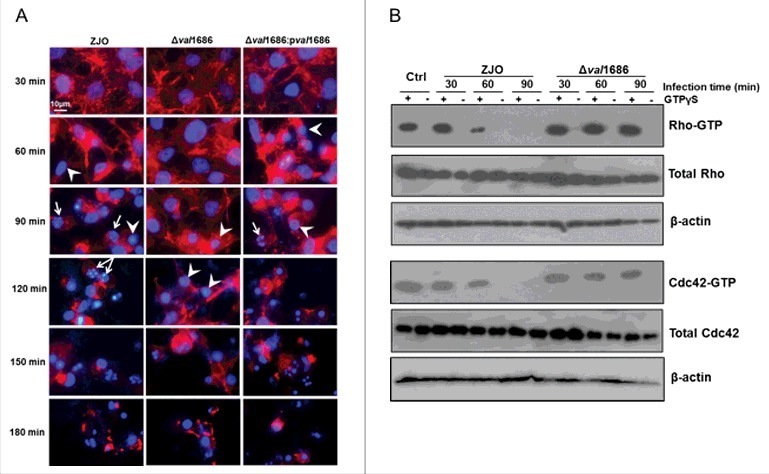
**Val1686 contributes to the delay of cell rounding and nuclear fragmentation, and the inactivation of Rho family GTPase in fish cells during the *V. alginolyticus* infection. (A)** FHM cells were infected with ZJO, ZJOΔ*val1686* (Δ*val1686*) and ZJOΔ*val1686*:p*val1686* (Δ*val1686*:p*val1686*). Cells were visualized under a fluorescence microscopy using rhodamine phalloidin to stain actin (red) and Hoechst to stain nuclei (blue) at indicated time points after infection. Arrows indicate fragmented nuclei (apoptotic bodies), while arrowheads indicate condensed nuclei. Scale bar: 10 µm. **(B)** FHM cells were infected with ZJO and ZJOΔ*val1686* (Δ*val1686*) and cell lysate were prepared as described in Materials and Methods. The lysates were incubated with (+) or without (-) GTPγS as indicated, followed by GTPases pulldown assays. Active Rho or Cdc42 (GTP-bound) were detected using corresponding antibodies. Upper panels indicate active GTPase, while middle panels represent total cellular GTPase, which were detected from total cleared lysates. Lower panels represent β-actin loading controls. Uninfected FHM cells were included as controls (Ctrl) in this assay.

**Val1686 contributes to *V. alginolyticus* T3SS-mediated cell rounding in fish cells**. Seven putative T3SS-related genes were predicted through the comparative genome analysis in *V. alginolyticus* and five of them share high sequence identity to these well-characterized T3SS1 effector-encoding genes in *V. parahaemolyticus* strain RIMD2210633 (Fig. S1). *val1686* and *val1680* from *V. alginolyticus* are putative orthologues for *vp1686* (VopS; 91% amino acid identity) and *vp1680* (VopQ; 88% amino acid identity) from *V. parahaemolyticus*, respectively. To determine the functional activity of Val1686 and Val1680, the deletion mutants and corresponding complementation strains were generated (Fig. S2). FHM cells were infected with either wild-type *V. alginolyticus* or deletion mutants and the infection was monitored over a 3 h time course. Cell rounding was observed for both wild-type strain (ZJO; 60min) and *val1686* deletion mutant (Δ*val1686*; 90min), but the deletion of the *val1686* gene resulted in a measurable delay (∼30 min) in this phenotype (Fig. 1A and Fig. S3). Extensive cell lysis was found by the end of both infections (Fig. 1A and Fig. S3). *In trans* expression of *val1686* successfully restored the earlier cell rounding phenotype (Fig. 1A). As a control, uninfected cells exhibited normal actin cytoskeleton and nucleus phenotypes (Fig. S4).

VopS of *V. parahaemolyticus* was implicated in cell rounding by inhibiting Rho family of guanosine triphosphatases (GTPases) [14,15]. In a similar manner, we found that the Val1686-mediated cell rounding involves interference with activation of Rho GTPases in *V. alginolyticus* (Fig. 1B). This was suggested from a GTPase pulldown assay showing that active RhoA and Cdc42 were no longer detected in ZJO-infected cell lysates after 1 h of infection, even if they were stimulated with the nonhydrolyzable GTP analogue GTPγS (Fig. 1B). In contrast, active RhoA and Cdc42 were still detected in cells infected with the *val1686* deletion mutant (Fig. 1B). These results reveal that Val1686 in *V. alginolyticus*, like VopS in *V. parahaemolyticus* for mammal cells, contributes to T3SS-mediated cell rounding in fish cells.

**Figure 2.**
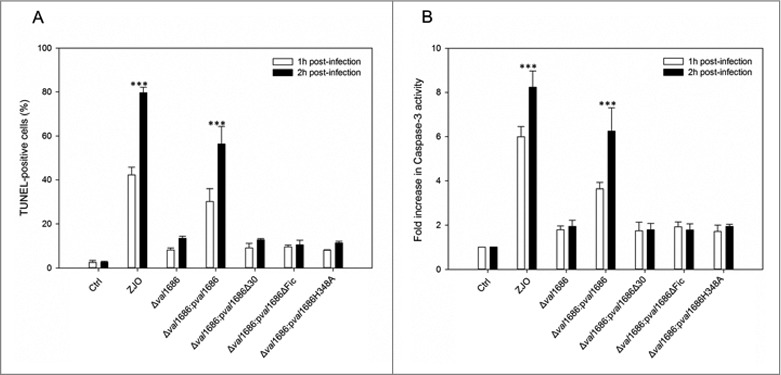
Val1686 is required for *V. alginolyticus* T3SS-induced apoptosis. (A) TUNEL assay. FHM cells were either uninfected, or infected (m.o.i. = 10) with wild-type ZJO (WT), deletion mutant and complementation strains as indicated. At 1 h and 2 h after infection, DNA fragmentation was detected by TUNEL staining. TUNEL-positive cells were quantified from three independent experiments and the data are presented as means ± SEM. At least four fields under light microscopy (40 × objective) were randomly selected and examined for each experiment. (B) Measurement of caspase-3 activity of infected cells as described for TUNEL assay. The data are expressed as fold-increase compared to the corresponding values of caspase activity in uninfected cells (Ctrl) (error bars = SEM; 3 independent experiments). Statistical analysis was performed by using a Kruskal Wallis test followed by a Tukey-Kramer multiple comparisons test between the treatment groups and the control. Asterisks indicate significance for both 1- and 2-hour time points compared to controls (***; *P* < 0.001).

**Val1686 is required and sufficient for *V. alginolyticus* T3SS-induced apoptosis**. Apoptotic bodies were observed after 90 min infection with the ZJO strain (Fig. 1A). By contrast, apoptotic bodies were largely absent for cells that were infected with the Δ*val1686* strain, although nuclear condensation was evident (Fig. 1A). TUNEL assays indicated that after 2 h, ∼80% of ZJO-infected FHM were positive for nuclear fragmentation whereas only ∼15% were positive for Δ*val1686*-infected cells (Fig. 2A and Fig. S5). Furthermore, caspase-3 in ZJO-infected cells was activated (present in the form of a cleaved procaspase-3) and its activity reached levels approximately 6 to 8-fold higher than those in control cells at 1 h and 2 h post infection (Fig. 2B). Caspase-3 activity from Δ*val1686*-infected cells was much less pronounced (Fig. 2B) while complementation restored ∼75% of the wild-type fragmentation and caspase-3 phenotypes (Fig. 2A and Fig. 2B, respectively). These data are consistent with Val1686 playing a key role in *V. alginolyticus* T3SS-induced apoptosis.

**Figure 3.**
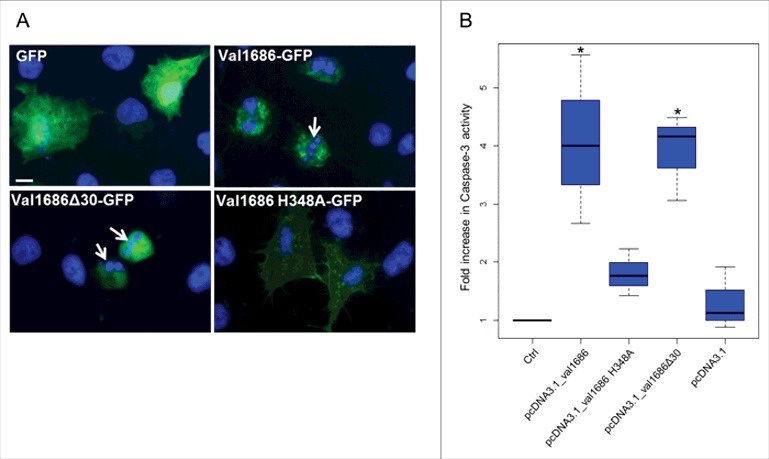
Val1686 alone is sufficient to induce apoptosis in transfected cells. (A) FHM cells were transfected with the plasmids pEGFP-N3, pEGFP_*val1686*, pEGFP_*val1686*Δ30 or pEGFP_*val1686* H348A and visualized with fluorescence microscopy. Green fluorescence shows GFP alone (as control), or GFP fusion proteins (Val1686-GFP, Val1686Δ30-GFP, Val1686H348A-GFP). Nuclei were stained by using Hoechst33258 (blue). Arrows indicate the fragmented nuclei. (B) FHM cells were respectively transfected with the plasmids pcDNA3.1, pcDNA_*val1686*, pcDNA_*val1686*Δ30 or pcDNA_*val1686* H348A and harvested for caspase-3 activity assay. The data are expressed as fold-increase compared to the corresponding values of caspase activity in uninfected cells (Ctrl) (3 independent experiments). (No statistical significance observed, *P* = 0.02)

To determine if Val1686 alone is sufficient to induce apoptosis, we transiently transfected FHM cells with a plasmid that encoded recombinant GFP solely or as a Val1686-GFP fusion protein. After 8 h the GFP-only transfected cells were positive for widely dispersed GFP in both the cytosol and nucleus (Fig. 3A). FHM cells transfected with the Val1686-GFP fusion protein exhibited cell rounding and nuclear fragmentation at 8 h after transfection (Fig. 3A). Caspase-3 activity was clearly detected in Val1686 transfected cells (Fig. 3B). Collectively, these data are consistent with Val1686 contributing to cell rounding, and with this protein being necessary and sufficient to induce apoptosis in FHM cells.

**Figure 4.**
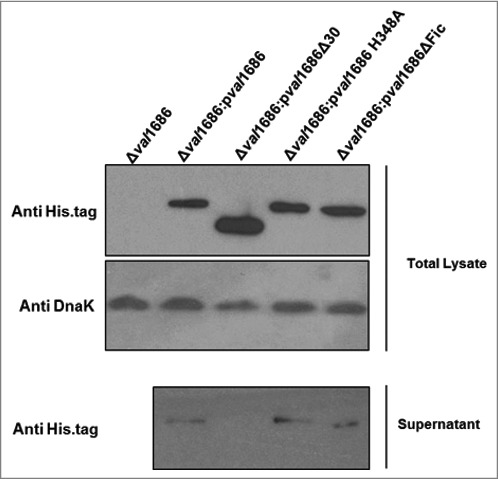
**Western blot analysis of Val1686 secretion**. Overnight cultures of Δ*val1686* strain and several complemented strains. Complementation included strains that synthesis native Val1686 (p*val1686*) or Val1686 without the first 30 amino acids (p*val1686*Δ30; negative control), or Val1686 having an amino acid change (histidine to alanine, position 348; p*val1686* H348A) or Val1686 lacking the Fic domain (p*val1686*ΔFic). Equal volume (10 μL) of prepared samples were loaded onto SDS-PAGE gel and the histidine tag in C-terminus was probed for individual recombinant protein in western blot. Anti-DnaK was used as a loading control. The data are representative of three repeated experiments.

**The Fic domain is necessary for apoptotic activity of Val1686**. Fic (filamentation induced by cAMP) domain is required for *V. parahaemolyticus* VopS-induced cell rounding [15]. Sequence alignment suggested that the Fic-conserved motif (HPFXXGNG) and the key H348 residue are present in Val1686 of *V. alginolyticus* (data not shown). To determine if the apoptotic activity of Val1686 is Fic domain-dependent, we complemented the Δ*val1686* mutant strain with either *val1686* lacking the HPFTDG region or with a version having a mutated residue H348A. Unlike complementation with the full-length, wild-type Val1686 protein (Fig. 1A), the apoptosis phenotype was not restored for either of these complemented strains (Δ*val1686*:p*val1686*ΔFic and Δ*val1686*:p*val1686* H348A), as evidenced by TUNEL and caspase-3 activity assays (Fig. S5 and, Fig. 2, respectively). Western blot analysis confirmed that the two mutants of Val1686, like wild-type Val1686 were present in the supernatant of their complemented strains (Fig. 4, Lane 3 and Lane 4 in lower panel), which also indicates that the Fic domain is not required for secretion of Val1686. As a control, another mutant of Val1686, which lacked the first 30 amino acid residues, was complemented into the Δ*val1686* (Δ*val1686*:p*val1686*Δ30). Although the mutated Val1686 is synthesized in the complemented strain (Fig. 4, Lane 3, upper panel), it was not present in the supernatant of Δ*val1686*:p*val1686*Δ30 strain (Fig. 4, Lane 2, lower panel), suggesting that the first 30 amino acid residues are essential to secrete the effector Val1686 during infection. As expected, the Δ*val1686*:p*val1686*Δ30 strain did not induce apoptosis based on TUNEL and caspase-3 activity assays (Fig. S5 and Fig. 2).

The above observation was also supported by a transfection assay. When the histidine residue was changed to alanine in the Val1686-GFP fusion protein (Val1686 H348A-GFP) or for the Val1686 without GFP (Val1686 H348A), apoptosis did not occur as indicated by a lack of change in cell morphology and Capase-3 activity of transfected FHM cells (Fig. 3). By contrast, when FHM cells were transfected with the truncated Val1686 (Val1686Δ30-GFP or Val1686Δ30) cell rounding, nuclear fragmentation and caspase-3 activation were evident (Fig. 3); this result is also consistent with the secretion signal being located within the first 30 amino acid residues of Val1686. Results from gene knockout, complementation and transfection experiments are consistent with the Fic domain being necessary for Val1686 to induce apoptosis in FHM cells.

**Figure 5.**
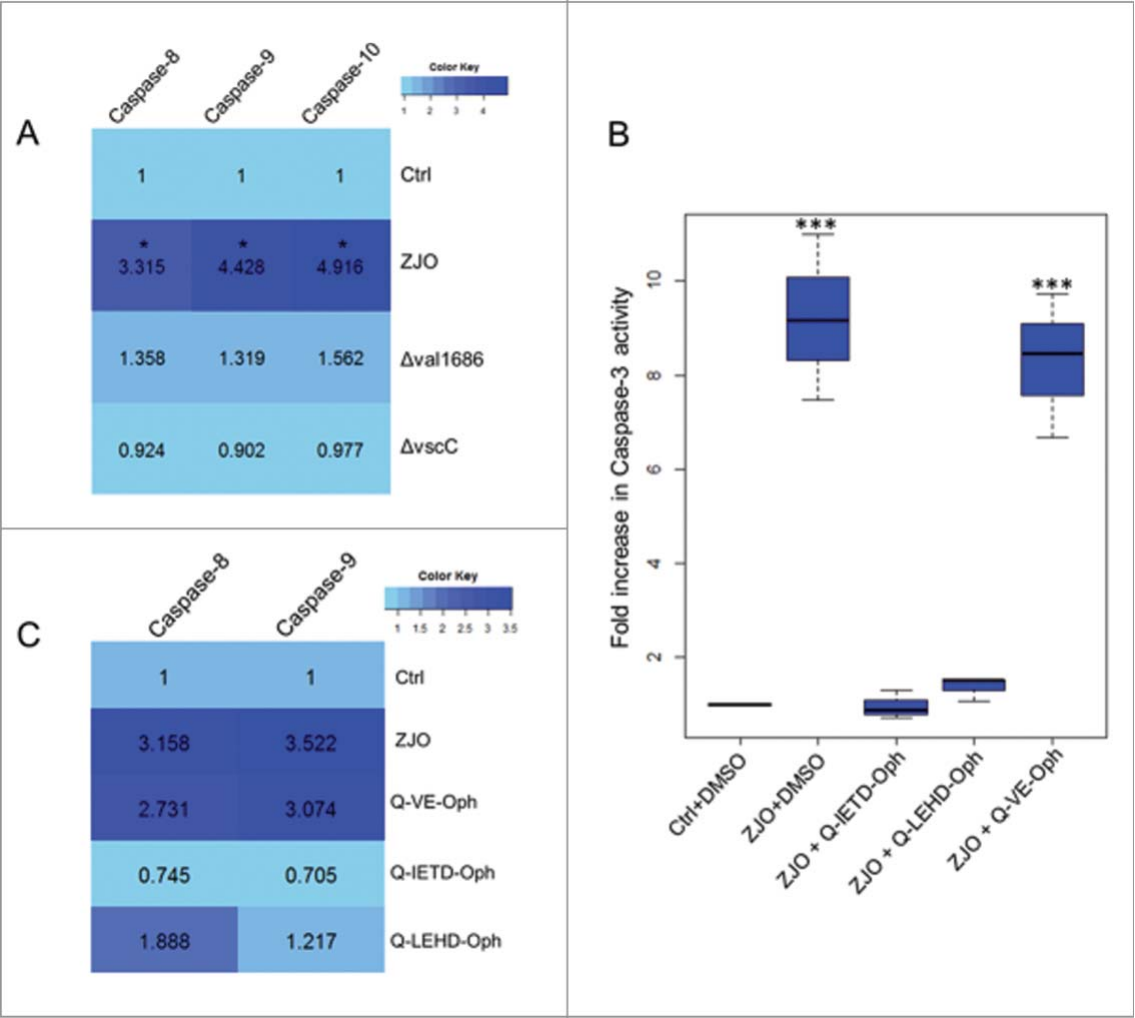
**Val1686 induced-apoptosis depends on caspases activation**. (A) Measurement of caspase-8, -9 and -10 activity. FHM cells were either uninfected (Ctrl), or infected with wild-type ZJO (WT), Δ*val1686* or Δ*vscC*. caspase-8, -9 and -10 activity assays were conducted after 2 h infection. (B) Inhibitory effect of three caspase inhibitors on *V. alginolyticus*-induced apoptosis. FHM cells were pre-incubated for 1 h with 20 μM of caspase-8 inhibitor (Q-IETD-Oph), caspase-9 inhibitor (Q-LEHD-Oph) and negative control inhibitor (Q-VE-Oph) and then infected with wild-type ZJO. Inhibitory effects on apoptosis were evaluated using the caspase-3 activity assay. (C) Inhibitory effect of three caspase inhibitors on caspase-8 and caspase-9 activity. FHM cells were treated as in (A) and measured for caspase-8 and -9 activity. The data are expressed as fold-increase compared to the corresponding values of caspase activity in uninfected cells (Ctrl) (3 independent experiments). A Kruskal Wallis test followed by a Tukey-Kramer multiple comparisons test versus control group (* *P* < 0.05, *** *P* < 0.001).

**Val1686 induced-apoptosis depends on activation of caspase-8/10 and caspase-9 in infected fish cells**. Caspase-3 is an apoptotic effector caspase that is activated by the presence of Val1686 (see above). We next investigated activation of initiator caspases (caspase-8/10 and caspase-9). Activation of caspase-8, -9, and -10 was observed in the wild-type ZJO-infected cells after 2 h of infection, while the Δ*val1686* strain was unable to induce these initiator caspases. For this experiment a T3SS-dysfunctional strain (Δ*vscC*) was included as a negative control (Fig. 5A). We treated FHM cells with inhibitors against caspase-8 and -9 (Q-IETD-Oph and Q-LEHD-Oph) for 1 h prior to infection and then measured nuclear fragmentation and caspase-3 activity. Both Q-IETD-Oph and Q-LEHD-Oph blocked nuclear fragmentation and caspase-3 activation in ZJO-infected cells (Q-VE-Oph served as a negative control for these experiments (Fig. 5B and Fig. S6). When FHM cells were pretreated with caspase inhibitors (1 h), Q-IETD-Oph suppressed caspase-8 and caspase-9 activation, while Q-LEHD-Oph inhibited caspase-9 activation and had less effect on caspase-8 (Fig. 5C). Taken together this data indicates that Val1686 induced-apoptosis depends on activation of caspases (caspase-3, -8, -9 and -10) in infected fish cells.

**Figure 6.**
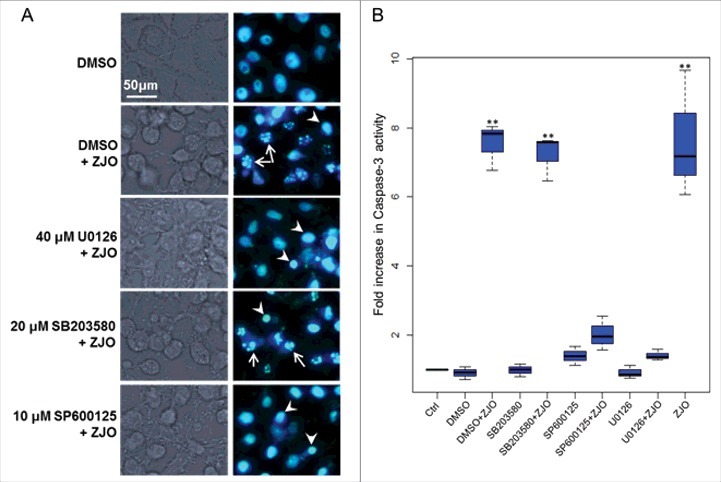
**Activated ERK and JNK pathways are involved in *V. alginolyticus*-induced apoptosis**. FHM cells were pre-incubated with 40 μM of ERK inhibitor (U0126-EtOH), 20 μM of p38 MAPK inhibitor (SB203580) and 10 μM of JNK inhibitor (SP600125) as described in the Materials and Methods. Inhibitory effect on *V. alginolyticus*-induced apoptosis was evaluated using Hoechst33258 staining (A) and caspase-3 activity assay (B). Arrows indicate the fragmented nuclei, while arrowheads indicate condensed nuclei. Scale bar = 50 µm. The data are expressed as fold-increase compared to the corresponding values of caspase activity in uninfected cells (Ctrl) (3 independent experiments). A Kruskal Wallis test followed by a Tukey-Kramer multiple comparisons test versus control group (** *P* < 0.01).

**ERK and JNK pathways are important for Val1686-induced apoptosis**. Mitogen-activated protein kinase (MAPK) signaling pathways are targeted by various bacterial type III effectors that triggers different cellular responses [39,40]. To confirm whether the MAPK (ERK, p38 and JNK) pathway is involved in the Val1686-induced apoptosis, FHM cells were treated with three kinase inhibitors (U0126, SB203580 or SP00125) for 1 h prior to bacterial infection. The concentration of the inhibitors used did not affect cell viability until 8 h (data not shown). None of the inhibitors blocked ZJO-induced cell rounding, but U0126 and SP00125 almost completely prevented the ZJO-infected cells from undergoing nuclear fragmentation after 2 h of infection (Fig. 6A). In contrast, SB203580-treated cells infected with ZJO still displayed nuclear fragmentation (Fig. 6A; compare with DMSO+ZJO infected cells). Consistent with the DNA staining results, U0126 and SP00125, but not SB203580, repressed activation of caspase-3 in the ZJO-infected cells after 2 h of infection (Fig. 6B). Collectively, these data are consistent with *V. alginolyticus* infection activating the JNK and ERK pathways and this activation is involved with Val1686-induced apoptosis in FHM cells.

**Figure 7.**
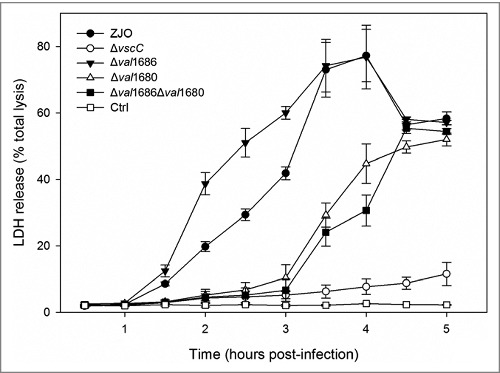
**Deletion of val1680 reduced *V. alginolyticus* T3SS-induced LDH release**. FHM cells were either uninfected or infected with *V. alginolyticus* strains ZJO, Δ*val1686*, Δ*val1680*, Δ*val1686*Δ*val1680* and Δ*vscC* as described in the Materials and Methods. At the indicated time points, culture supernatants were measured for the release of LDH and calculated as a percentage of total cellular lysis. The data are expressed as means ± SEM from three independent experiments.

**Val1680, not Val1686, plays a role in *V. alginolyticus* T3SS-induced LDH release**. T3SS of *V. alginolyticus* induces three different events (apoptosis, cell rounding and osmotic lysis) in infected fish cells (Zhao et al., 2010) [32]. Val1680 is a putative orthologue of VP1680 (VopQ) of *V. parahaemolyticus*, and the latter is not only responsible for autophagy induction, but also contributes to release of cellular contents in *V. parahaemolyticus-*infected HeLa cells (Burdette et al., 2009) [10]. To determine if Val1686 or Val1680 contributes to an osmotic lysis phenotype, we measured LDH release of infected cells by the *V. alginolyticus* ZJO, Δ*val1686*, Δ*val1680*, and Δ*val1686*Δ*val1680* strains over a 5 h time course. Deletion of *val1686* did not reduce LDH release (Fig. 7). In contrast, deletion of *val1680* resulted in delayed release of LDH (Fig. 7). Complementation of the *val1680* restored the ability of the Δ*val1680* strain to efficiently cause the phenotype of LDH release (Fig. S7A) while deletion of *val1680* from *V. alginolyticus* did not have any observable effect on T3SS-mediated cell rounding and apoptosis (Fig. S3). A Δ*val1686*Δ*val1680* strain exhibited a similar delay in LDH release as the Δ*val1680* strain (Fig. 7). FHM cells infected with the double-knockout strain for 5 h still maintained a uniform monolayer and the cell appeared similar to that of the Δ*vscC*-infected cells (Fig. S7B). Based on these observations, we surmise that Val1680 contributes to *V. alginolyticus* T3SS-induced LDH release, but unknown effector(s) may also be involved in this event.

## Discussion

Zhao et al. (2010) [32] showed that *in vitro V*. *alginolyticus* infection causes fish cells to undergo cell rounding, apoptosis and cell lysis. In this study, we further characterized the contribution of two *V. alginolyticus* T3SS effectors (Val1686 and Val1680) to these phenotypes. Val1686 is a Fic-domain containing protein that is a likely orthologue to VopS in *V. parahaemolyticus*. Val1686 appears to function in a similar manner to VopS, which induces cell rounding by inhibiting the Rho family of guanosine triphosphatases (GTPases), but it is also necessary and sufficient to trigger apoptosis in *V. alginolyticus*-infected fish cells. Studies suggests that Rho GTPases have other functions including effects on gene transcription [41], cell cycle control [42], and cellular survival and death [43,44]. One orthologous effector to VopS in *Pseudomonas aeruginosa*, ExoS, modifies the Rho GTPases through ADP-ribosylating activity, and this modification is required for induction of apoptosis in host cells [45,46]. Consequently, targeting Rho GTPases may lead to different phenotypes [47−49], and therefore is not surprising to find that the Val1686 can evoke two distinct phenotypes (cell rounding and apoptosis) in fish cells. This conclusion is supported by our assays for the apoptotic activity of Fic domain in Val1686. We demonstrated that the Fic domain is not only necessary for cell rounding, but it is required for the apoptotic phenotype. This is consistent with a previous study in *V. parahaemolyticus*, where the Fic domain was responsible for collapse of the actin cytoskeleton by AMPylation of Rho GTPases by VopS, which induces cell rounding [15]. Consequently, we infer that the inactivation of Rho GTPases by Val1686 concurrently evokes two different events (cell rounding and apoptosis) in *V. alginolyticus* infected fish cells.

Apoptosis is mediated by a family of cysteine-dependent, aspartate-specific proteases known as caspases [50,51]. The caspase family can be divided into two functional subgroups: initiator and effector caspases. The initiator caspases (caspase-2, -8, -9, -10) work to auto-activate and initiate the proteolytic processing of other caspases in response to apoptotic stimuli, while effector caspases (caspase-3, -6, -7) are activated by initiator caspases and are primarily responsible for the dismantling of the apoptotic cell [52]. As expected, activated caspase-3 was detected in the Val1686-induced apoptosis of FHM cells. We did not expect, however, to observe simultaneous activation of caspase-8, -9 and -10 because caspase-8 and -10 are involved in an extrinsic (receptor-mediated) pathway while caspase-9 is involved in an intrinsic (mitochondria-mediated) pathway of apoptosis in mammals [53]. Furthermore, we found evidence that caspase-8 is activated upstream of caspase-9 in the Val1686-induced apoptosis of fish cells. Normally, caspase-8 is activated in a receptor-mediated pathway after which it cleaves and activates caspase-3 [53]. In some situations, activated caspase-8 can also initiate the crosstalk between the extrinsic and intrinsic pathways by cleaving the BH3-only protein, Bid, and then the truncated Bid (tBid) is translocated to mitochondria. This promotes cytochrome C release, which subsequently activates caspase-9 and -3 [54]. Our findings suggest that Val1686 activation of caspase-8 is responsible for activation of caspase-9 by evoking cytochrome C release, which subsequently results in the activation of caspase-3 in fish cells. Unfortunately, existing reagents for detecting cytochrome C and Bid are optimized for mammalian systems and are not able to detect these proteins in fish cells. Nevertheless, these data are consistent with Val1686-induced activation of caspase-8/-10, -9 and -3 in a cascade-like manner, leading to apoptosis in fish cells.

The MAPK are a group of protein serine/threonine kinases that induce different cellular responses upon extracellular stimuli [55] and three main types of MAPK signaling pathways have been characterized to date [56]. The ERK1/2 pathway is important for cell proliferation and differentiation, while the JNK and p38 pathways are deemed stress responsive and thus involved in cell survival [56,57]. Pathogenic bacteria commonly manipulate these pathways via an extensive list of T3SS effector proteins [39,40,50]. For example, Woolery et al (2014) reported that AMPylation of Rho GTPases by VopS inhibited the activation of JNK and ERK signaling pathways during infection with *V. parahaemolyticus*. Herein, we show that the activation of ERK and JNK pathways are required for Val1686-induced apoptosis of *V. alginolyticus* in fish cells. Given that stress signals are usually transmitted to the MAPK signaling cascades by Rho family GTPases [58], we infer that Rho GTPases inactivation caused by effector Val1686 of *V. alginolyticus* T3SS further stimulates apoptosis via activation of ERK and JNK-dependent signaling pathways in FHM cells.

VopQ (Vp1680), is a T3SS1 effector from *V. parahaemolyticus* that contributes to induction of rapid autophagy in HeLa cells. This effector functions by interacting with the Vo domain of the conserved V-type H^+^-ATPase (V-ATPase) that forms a gated channel in lysosomal membranes [10,18,19]. Although the *V. alginolyticus* orthologue (Val1680) of effector VopQ is present, autophagy was not detected by monodansylcadaverine (MDC) staining [35], nor was LC3 conversion detected after FHM cells were infected with *V. alginolyticus* (data not shown). We are not able to determine the detailed mechanism behind this difference without additional experiments, but the inherent differences between fish and mammalian cells may contribute to this effect. Burdette et al., (2009) reported that deletion of VopQ attenuates the ability of *V. parahaemolyticus* to induce release of cellular contents in addition to autophagy, and we observed a similar event in *V. alginolyticus* by showing Val1680 contributes to T3SS-induced LDH release. We also speculate that other T3SS effector(s) of *V. alginolyticus* may also contribute to fish cells lysis because the Δ*val1686*Δ*val1680* strain was still capable of inducing lysis (albeit delayed) while a strain lacking a functional T3SS (Δ*vscC* strain) did not. VPA0450, another effector secreted by T3SS1 of *V. parahaemolyticus*, reportedly accelerates cell lysis by disrupting membrane integrity [22], but its homolog is not present in the genome of *V. alginolyticus*.

In summary, we describe the functional activity of two T3SS effectors from *V. alginolyticus* (Val1686 and Val1680) that are responsible for the *in vitro* death of FHM cells. Val1686 not only mediates cell rounding by inhibiting Rho family GTPases, but it also activates the apoptosis pathway via Val1686-dependent GTPase inhibition. Subsequently, this effector activates the ERK and JNK signaling pathways and caspase-8/-10, -9 and -3 in a cascade-like manner, thereby leading to an apoptotic phenotype in fish cells. The other effector protein, Val1680, was found to contribute to T3SS-dependent lysis of fish cells which behaves differently from its orthologue, VopQ which induces autophagy in *V. parahaemolyticus*. Together, we report here that Val1686 and Val1680 are involved in the induction of apoptosis, cell rounding and cell lysis in *V. alginolyticus*-infected fish cells which certainly aid in the understanding of *Vibrio* pathogenesis.

## Supplementary Material

KVIR_I_141314.zip

## References

[1] Galan JE, Wolf-Watz H Protein delivery into eukaryotic cells by type III secretion machines. Nature. 2006;444:567–73. doi:10.1038/nature05272 17136086

[2] Mueller CA, Broz P, Cornelis GR The type III secretion system tip complex and translocon. Mol Microbiol. 2008;68:1085–95. doi:10.1111/j.1365-2958.2008.06237.x 18430138

[3] Enninga J, Rosenshine I Imaging the assembly, structure and activity of type III secretion systems. Cell Microbiol. 2009;11:1462–70. doi:10.1111/j.1462-5822.2009.01360.x 19622097

[4] Hueck CJ. Type III protein secretion systems in bacterial pathogens of animals and plants. Microbiol Mol Biol Rev. 1998;62:379–433. 9618447 10.1128/mmbr.62.2.379-433.1998 PMC98920

[5] Coburn B, Sekirov I, Finlay BB Type III secretion systems and disease. Clin Microbiol Rev. 2007;20:535–49. doi:10.1128/CMR.00013-07 17934073 PMC2176049

[6] Troisfontaines P, Cornelis GR Type III secretion: more systems than you think. Physiology (Bethesda). 2005;20:326–39. doi:10.1152/physiol.00011.2005 16174872

[7] Michiels T, Wattiau P, Brasseur R, Ruysschaert JM, Cornelis G Secretion of Yop proteins by Yersiniae. Infect Immun. 1990; 58:2840–9. 2129533 10.1128/iai.58.9.2840-2849.1990 PMC313576

[8] Makino K, Oshima K, Kurokawa K, Yokoyama K, Uda T, Tagomori K, et al. Genome sequence of Vibrio parahaemolyticus: a pathogenic mechanism distinct from that of V cholerae. Lancet. 2003;361:743–9. doi:10.1016/S0140-6736(03)12659-1 12620739

[9] Park KS, Ono T, Rokuda M, Jang MH, Okada K, Iida T, et al. Functional characterization of two type III secretion systems of Vibrio parahaemolyticus. Infect Immun. 2004;72:6659–65. doi:10.1128/IAI.72.11.6659-6665.2004 15501799 PMC523034

[10] Burdette DL, Seemann J, Orth K Vibrio VopQ induces PI3-kinase-independent autophagy and antagonizes phagocytosis. Mol Microbiol. 2009;73:639–49. doi:10.1111/j.1365-2958.2009.06798.x 19627496 PMC2733864

[11] Zhou X, Konkel ME, Call DR Type III secretion system 1 of Vibrio parahaemolyticus induces oncosis in both epithelial and monocytic cell lines. Microbiology. 2009;155:837–51. doi:10.1099/mic.0.024919-0 19246755

[12] Pineyro P, Zhou X, Orfe LH, Friel PJ, Lahmers K, Call DR Development of two animal models to study the function of Vibrio parahaemolyticus type III secretion systems. Infect Immun. 2010;78:4551–9. doi:10.1128/IAI.00461-10 20823199 PMC2976342

[13] Ritchie JM, Rui H, Zhou X, Iida T, Kodoma T, Ito S, et al. Inflammation and disintegration of intestinal villi in an experimental model for Vibrio parahaemolyticus-induced diarrhea. PLoS Pathog. 2012;8:e1002593. doi:10.1371/journal.ppat.1002593 22438811 PMC3305451

[14] Casselli T, Lynch T, Southward CM, Jones BW, DeVinney R Vibrio parahaemolyticus inhibition of Rho family GTPase activation requires a functional chromosome I type III secretion system. Infect Immun. 2008;76:2202–11. doi:10.1128/IAI.01704-07 18347050 PMC2346677

[15] Yarbrough ML, Li Y, Kinch LN, Grishin NV, Ball HL, Orth K AMPylation of Rho GTPases by Vibrio VopS disrupts effector binding and downstream signaling. Science. 2009;323:269–72. doi:10.1126/science.1166382 19039103

[16] Hall A. Rho GTPases and the actin cytoskeleton. Science 1998; 279:509–14. doi:10.1126/science.279.5350.509 9438836

[17] Wojnacki J, Quassollo G, Marzolo MP, Caceres A Rho GTPases at the crossroad of signaling networks in mammals: impact of Rho-GTPases on microtubule organization and dynamics. Small GTPases. 2014;5:e28430. doi:10.4161/sgtp.28430 24691223 PMC4114925

[18] Matsuda S, Okada N, Kodama T, Honda T, Iida T A cytotoxic type III secretion effector of Vibrio parahaemolyticus targets vacuolar H+-ATPase subunit c and ruptures host cell lysosomes. PLoS Pathog. 2012;8:e1002803. doi:10.1371/journal.ppat.1002803 22829766 PMC3400558

[19] Sreelatha A, Bennett TL, Carpinone EM, O'Brien KM, Jordan KD, Burdette DL, et al. Vibrio effector protein VopQ inhibits fusion of V-ATPase-containing membranes. Proc Natl Acad Sci U S A. 2015;112:100–5. doi:10.1073/pnas.1413764111 25453092 PMC4291640

[20] Salomon D, Guo Y, Kinch LN, Grishin NV, Gardner KH, Orth K Effectors of animal and plant pathogens use a common domain to bind host phosphoinositides. Nat Commun. 2013;4:2973. doi:10.1038/ncomms3973 24346350 PMC4981085

[21] Ono T, Park KS, Ueta M, Iida T, Honda T Identification of proteins secreted via Vibrio parahaemolyticus type III secretion system 1. Infect Immun. 2006;74:1032–42. doi:10.1128/IAI.74.2.1032-1042.2006 16428750 PMC1360304

[22] Broberg CA, Zhang L, Gonzalez H, Laskowski-Arce MA, Orth K A Vibrio effector protein is an inositol phosphatase and disrupts host cell membrane integrity. Science. 2010;329:1660–2. doi:10.1126/science.1192850 20724587

[23] Thompson CC, Vicente AC, Souza RC, Vasconcelos AT, Vesth T, Alves N Jr., et al. Genomic taxonomy of Vibrios. BMC Evol Biol. 2009;9:258. doi:10.1186/1471-2148-9-258 19860885 PMC2777879

[24] Nicholas A, Daniels AS A review of pathogenic vibrio infections for clinicians. Infect Med. 2000;17(10):665–85.

[25] Austin B. Vibrios as causal agents of zoonoses. Vet Microbiol. 2010;140:310–7. doi:10.1016/j.vetmic.2009.03.015 19342185

[26] Xie ZY, Hu CQ, Chen C, Zhang LP, Ren CH Investigation of seven Vibrio virulence genes among Vibrio alginolyticus and Vibrio parahaemolyticus strains from the coastal mariculture systems in Guangdong, China. Lett Appl Microbiol. 2005;41:202–7. doi:10.1111/j.1472-765X.2005.01688.x 16033522

[27] Chen MX, Li HY, Li G, Zheng TL Distribution of Vibrio alginolyticus-like species in Shenzhen coastal waters, China. Braz J Microbiol. 2011;42:884–96. doi:10.1590/S1517-83822011000300007 24031704 PMC3768764

[28] Lee KK, Yu SR, Liu PC Alkaline serine protease is an exotoxin of Vibrio alginolyticus in kuruma prawn, Penaeus japonicus. Curr Microbiol 1997; 34:110–7. doi:10.1007/s002849900153 9003588

[29] Zanetti S, Deriu A, Volterra L, Falchi MP, Molicotti P, Fadda G, et al. Virulence factors in Vibrio alginolyticus strains isolated from aquatic environments. Ann Ig. 2000;12:487–91. 11235505

[30] Gonzalez-Escalona N, Blackstone GM, DePaola A Characterization of a Vibrio alginolyticus strain, isolated from Alaskan oysters, carrying a hemolysin gene similar to the thermostable direct hemolysin-related hemolysin gene (trh) of Vibrio parahaemolyticus. Appl Environ Microbiol. 2006;72:7925–9. doi:10.1128/AEM.01548-06 17056701 PMC1694234

[31] Wang Q, Liu Q, Cao X, Yang M, Zhang Y Characterization of two TonB systems in marine fish pathogen Vibrio alginolyticus: their roles in iron utilization and virulence. Arch Microbiol. 2008;190:595–603. doi:10.1007/s00203-008-0407-1 18629473

[32] Zhao Z, Chen C, Hu CQ, Ren CH, Zhao JJ, Zhang LP, et al. The type III secretion system of Vibrio alginolyticus induces rapid apoptosis, cell rounding and osmotic lysis of fish cells. Microbiology. 2010;156:2864–72. doi:10.1099/mic.0.040626-0 20576689

[33] Milton DL, O'Toole R, Horstedt P, Wolf-Watz H Flagellin A is essential for the virulence of Vibrio anguillarum. J Bacteriol 1996; 178:1310–9. doi:10.1128/jb.178.5.1310-1319.1996 8631707 PMC177804

[34] Nydam SD, Shah DH, Call DR Transcriptome analysis of Vibrio parahaemolyticus in type III secretion system 1 inducing conditions. Front Cell Infect Microbiol. 2014;4:1. doi:10.3389/fcimb.2014.00001 24478989 PMC3895804

[35] Zhao Z, Zhang L, Ren C, Zhao J, Chen C, Jiang X, et al. Autophagy is induced by the type III secretion system of Vibrio alginolyticus in several mammalian cell lines. Arch Microbiol. 2011;193:53–61. doi:10.1007/s00203-010-0646-9 21046072

[36] Zhao Z, Orfe LH, Liu J, Lu SY, Besser TE, Call DR Microcin PDI regulation and proteolytic cleavage are unique among known microcins. Sci Rep. 2017;7:42529. doi:10.1038/srep42529 28205647 PMC5311971

[37] Liu J, Lu SY, Orfe LH, Ren CH, Hu CQ, Call DR, et al. ExsE Is a Negative Regulator for T3SS Gene Expression in Vibrio alginolyticus. Front Cell Infect Microbiol. 2016;6:177. doi:10.3389/fcimb.2016.00177 27999769 PMC5138213

[38] Zhou X, Konkel ME, Call DR Vp1659 is a Vibrio parahaemolyticus type III secretion system 1 protein that contributes to translocation of effector proteins needed to induce cytolysis, autophagy, and disruption of actin structure in HeLa cells. J Bacteriol. 20 10;192:3491–502. 20418402 10.1128/JB.01493-09 PMC2897656

[39] Bhavsar AP, Guttman JA, Finlay BB Manipulation of host-cell pathways by bacterial pathogens. Nature. 2007;449:827–34. doi:10.1038/nature06247 17943119

[40] Shan L, He P, Sheen J Intercepting host MAPK signaling cascades by bacterial type III effectors. Cell Host Microbe. 2007;1:167–74. doi:10.1016/j.chom.2007.04.008 18005696

[41] Benitah SA, Valeron PF, van Aelst L, Marshall CJ, Lacal JC Rho GTPases in human cancer: an unresolved link to upstream and downstream transcriptional regulation. Biochim Biophys Acta. 2004;1705:121–32. 15588766 10.1016/j.bbcan.2004.10.002

[42] Villalonga P, Ridley AJ Rho GTPases and cell cycle control. Growth Factors. 2006;24:159–64. doi:10.1080/08977190600560651 17191359

[43] Etienne-Manneville S, Hall A Rho GTPases in cell biology. Nature. 2002;420:629–35. doi:10.1038/nature01148 12478284

[44] Heasman SJ, Ridley AJ Mammalian Rho GTPases: new insights into their functions from in vivo studies. Nat Rev Mol Cell Biol. 2008;9:690–701. doi:10.1038/nrm2476 18719708

[45] Kaufman MR, Jia J, Zeng L, Ha U, Chow M, Jin S Pseudomonas aeruginosa mediated apoptosis requires the ADP-ribosylating activity of exoS. Microbiology. 2000;146 (Pt 10):2531–41. doi:10.1099/00221287-146-10-2531 11021928

[46] Jia J, Alaoui-El-Azher M, Chow M, Chambers TC, Baker H, Jin S c-Jun NH2-terminal kinase-mediated signaling is essential for Pseudomonas aeruginosa ExoS-induced apoptosis. Infect Immun. 2003;71:3361–70. doi:10.1128/IAI.71.6.3361-3370.2003 12761120 PMC155783

[47] Fiorentini C, Falzano L, Travaglione S, Fabbri A Hijacking Rho GTPases by protein toxins and apoptosis: molecular strategies of pathogenic bacteria. Cell Death Differ. 2003;10:147–52. doi:10.1038/sj.cdd.4401151 12700642

[48] Aktories K Bacterial protein toxins that modify host regulatory GTPases. Nat Rev Microbiol. 2011;9:487–98. doi:10.1038/nrmicro2592 21677684

[49] Popoff MR. Bacterial factors exploit eukaryotic Rho GTPase signaling cascades to promote invasion and proliferation within their host. Small GTPases. 2014;5:e983863. doi:10.4161/sgtp.28209. PMC4160336 25203748

[50] Raymond B, Young JC, Pallett M, Endres RG, Clements A, Frankel G Subversion of trafficking, apoptosis, and innate immunity by type III secretion system effectors. Trends Microbiol. 2013;21:430–41. doi:10.1016/j.tim.2013.06.008 23870533

[51] Taylor RC, Cullen SP, Martin SJ Apoptosis: controlled demolition at the cellular level. Nat Rev Mol Cell Biol. 2008;9:231–41. doi:10.1038/nrm2312 18073771

[52] Creagh EM, Conroy H, Martin SJ Caspase-activation pathways in apoptosis and immunity. Immunol Rev. 2003;193:10–21. doi:10.1034/j.1600-065X.2003.00048.x 12752666

[53] Li J, Yuan J Caspases in apoptosis and beyond. Oncogene. 2008;27:6194–206. doi:10.1038/onc.2008.297 18931687

[54] Kantari C, Walczak H Caspase-8 and bid: caught in the act between death receptors and mitochondria. Biochim Biophys Acta. 2011;1813:558–63. doi:10.1016/j.bbamcr.2011.01.026 21295084

[55] Chang L, Karin M Mammalian MAP kinase signalling cascades. Nature. 2001;410:37–40. doi:10.1038/35065000 11242034

[56] Johnson GL, Lapadat R Mitogen-activated protein kinase pathways mediated by ERK, JNK, and p38 protein kinases. Science. 2002;298:1911–2. doi:10.1126/science.1072682 12471242

[57] Wada T, Penninger JM Mitogen-activated protein kinases in apoptosis regulation. Oncogene. 2004;23:2838–49. doi:10.1038/sj.onc.1207556 15077147

[58] Kyriakis JM, Avruch J Mammalian MAPK signal transduction pathways activated by stress and inflammation: a 10-year update. Physiol Rev. 2012;92:689–737. doi:10.1152/physrev.00028.2011 22535895

