# Supplementary material for: The Vibrio alginolyticus T3SS effectors, Val1686 and Val1680, induce cell rounding, apoptosis and lysis of fish epithelial cells
Source: Virulence. 2018 Feb 27;9(1):318–30. doi: 10.1080/21505594.2017.1414134 (PMC5955196; doi:10.1080/21505594.2017.1414134)
Supplement: KVIR_I_141314.zip [file kvir-09-01-1414134-s001.zip › KVIR_I_141314/2017VIRULENCE0242R1-s02.docx]

**Figure S1. Genetic arrangement of the *val1686* and *val1680* in the effector-encoding region of T3SS in *V.alginolyticus* strain ZJO.** Each gene was represented by arrow indicating the approximate size and the direction of transcription based on the position of methionine initiation and termination codons. White arrows indicate the genes in the effector encoding region which is located between the structural genes *vsc*L and *vsc*U (indicated by black arrows). The effector-encoding region of T3SS1 in *V.parahaemolyticus* strain RIMD2210633 was included as reference. Homologous genes between *V.alginolyticus* and *V.parahaemolyticus* were connected by dashed line and amino acid identities of them were showed in parentheses below effector proteins of *V.alginolyticus.*


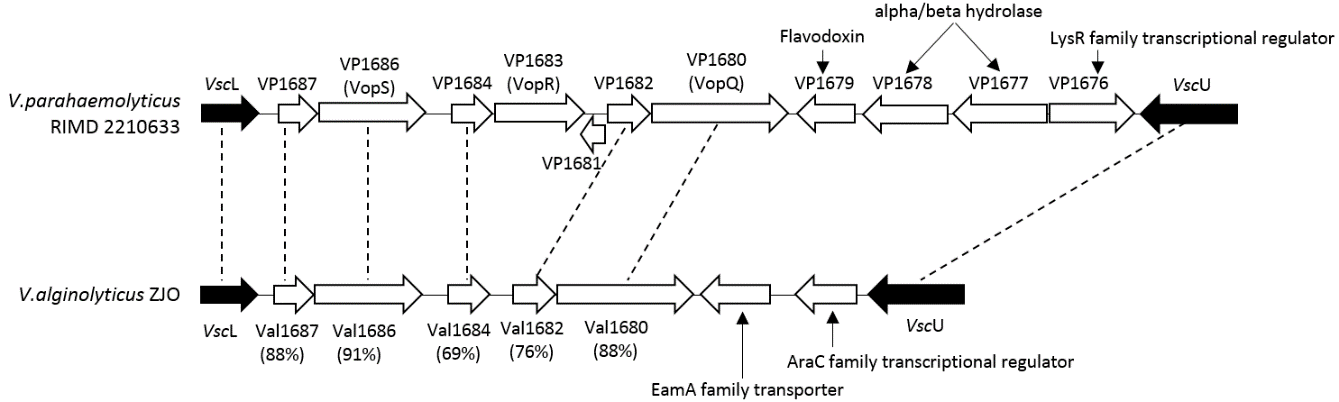


**Figure S2.** PCR confirmation for three deletion mutants of *V. alginolytiucs*. PCR was performed using primers located inside of the deleted sequence (val1686_int_F/R and val1680_int_F/R) with EmeraldAmp PCR Master Mix (Takara Biotechnology Co., Ltd) following to the manufacturer’s protocol. PCR products were resolved on agarose gel. Lane 1: wild-type ZJO, Lane 2: ZJOΔ*val1686*, Lane 3: ZJOΔ*val1680*, Lane 4: ZJOΔ*val1686*Δ*val1680*, M: DNA Marker DL2000 (from top to bottom: 2000, 1000, 750, 500, 250, 100 bp).

1 2 3 4 M 1 2 3 4

val1686_int_F/R

val1680_int_F/R

**
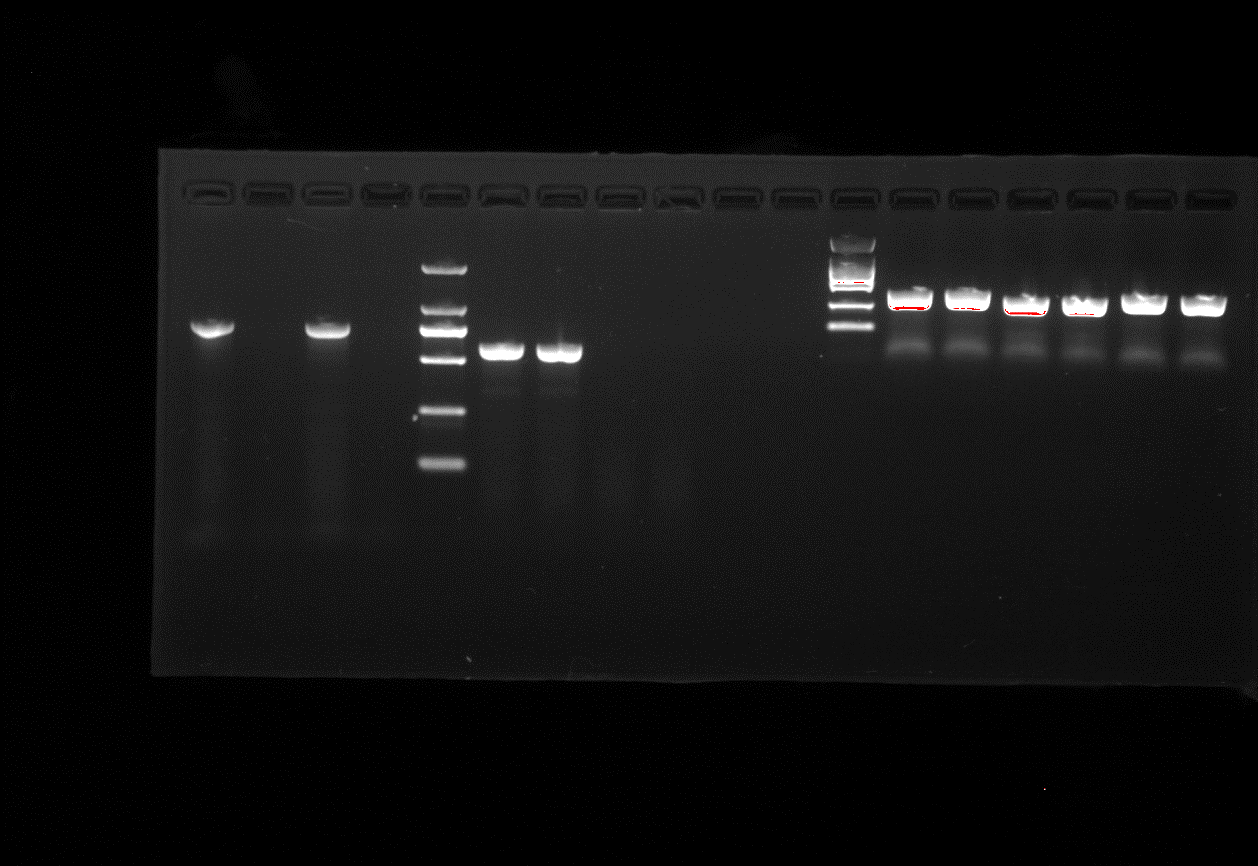
**

**Figure S3 Effects of Val1686 and Val1680 on *V. alginolyticus* T3SS-mediated cell rounding and nuclear fragmentation.** FHM cells were infected with wild-type ZJO, ZJOΔ*val1686* (Δ*val1686*), ZJOΔ*val1680* (Δ*val1680*) and ZJOΔ*val1686*Δ*val1680* (Δ*val1686*Δ*val1680*) as described in the Materials and Methods. Cells were fixed and stained by Hoechst at indicated time points after infection. Arrows indicate the fragmented nuclei, while arrowheads indicate condensed nuclei. Scale: 50 µm.


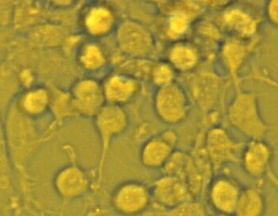

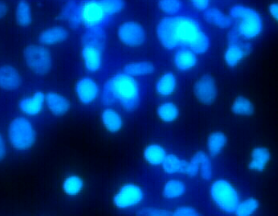

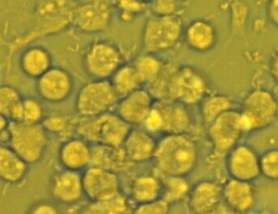

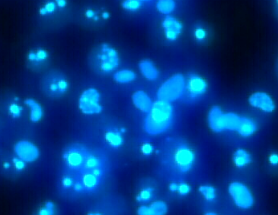

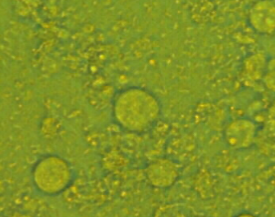

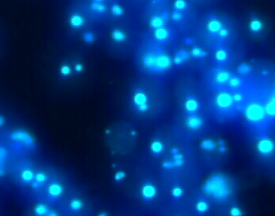

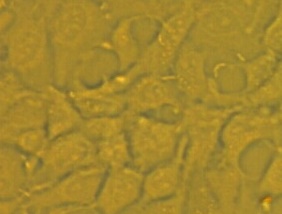

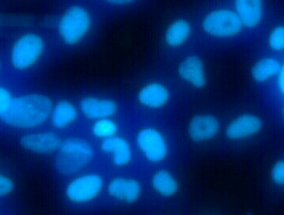

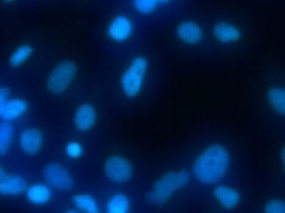

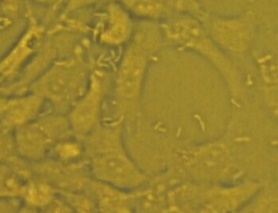

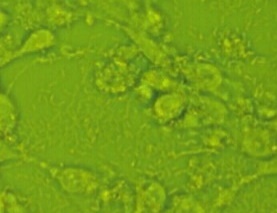

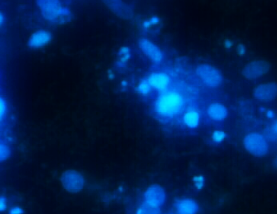

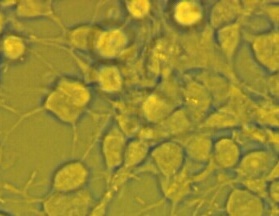

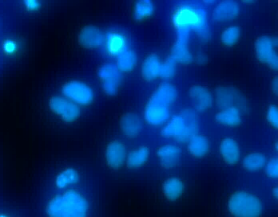

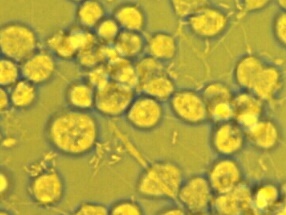

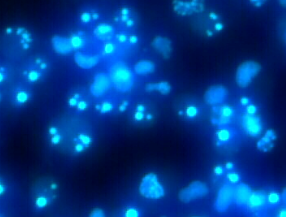

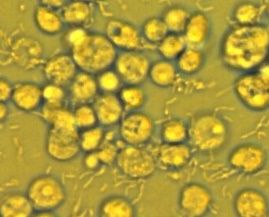

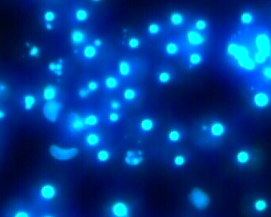

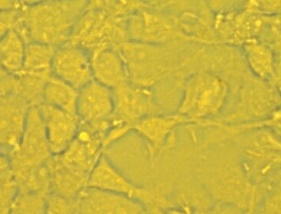

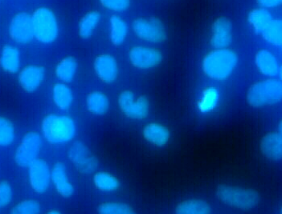

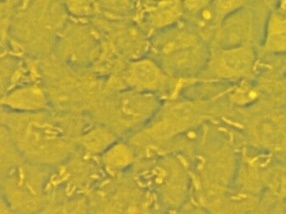

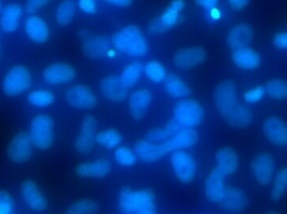

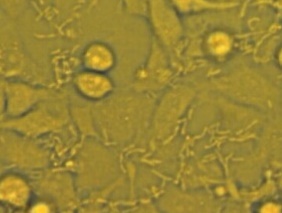

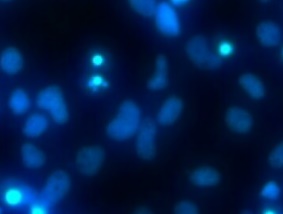


ZJO

Δ*val*1686

Δ*val*1680

Δ*val*1686 Δ*val*1680

1h

2h

3h

**Figure S4. Morphological observation of FHM cells after staining with rhodamine phalloidin and Hoechst.** The images serve as uninfected controls as part of Figure 1A, and red fluorescence indicates cytoskeleton and blue fluorescence indicates nuclei. Scale bar: 10 µm.


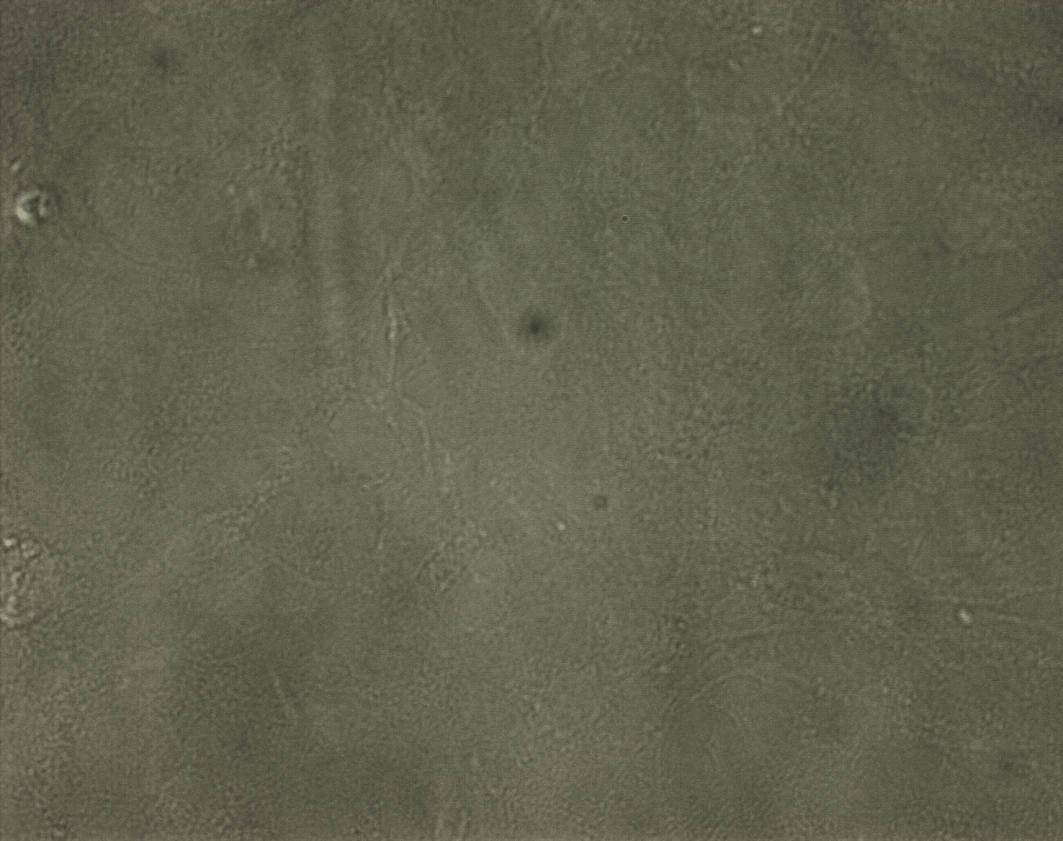

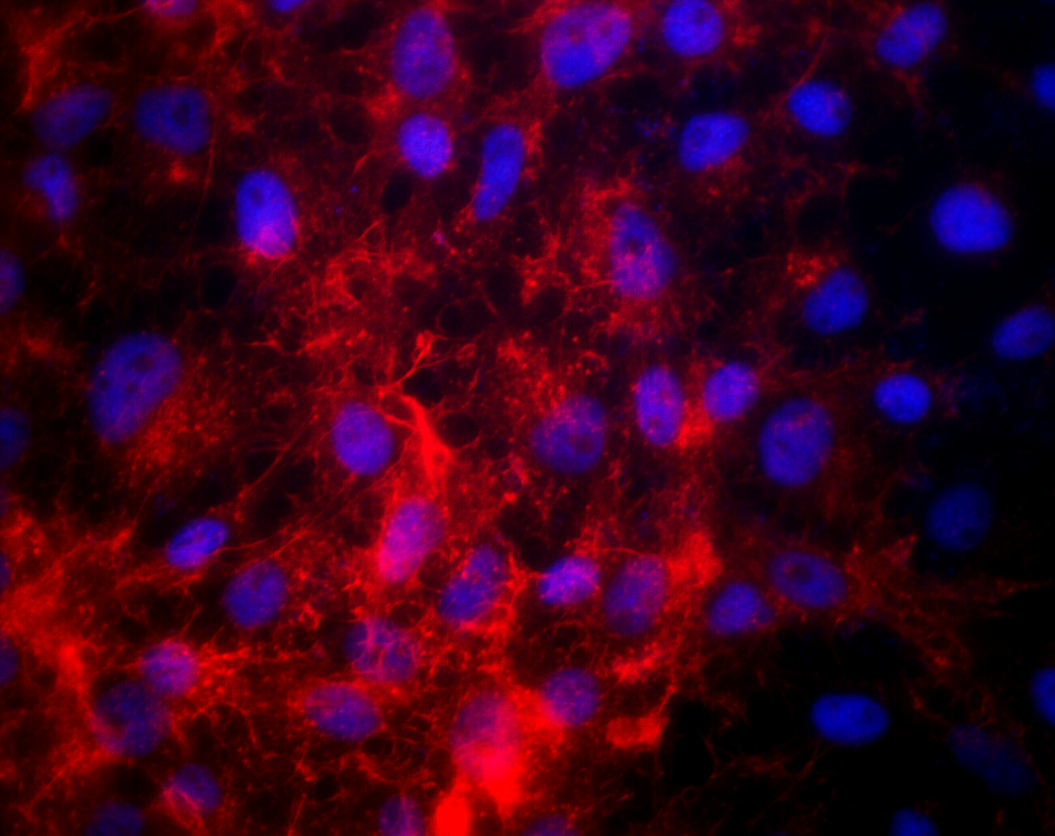


Light

Hoechst/ phalloidin

**Figure S5 Fluorescence microscopy of DNA fragmentation after TUNEL staining.** FHM cells were either uninfected (Ctrl), or infected with wild-type ZJO, deletion mutant and complementation strains as indicated. DNA fragmentation was detected by TUNEL method using the In situ Cell Death Detection kit, TMR red (Roche) after 2 h of infection. TUNEL-positive signal was observed under fluorescence microscopy. Scale bar: 50µm.


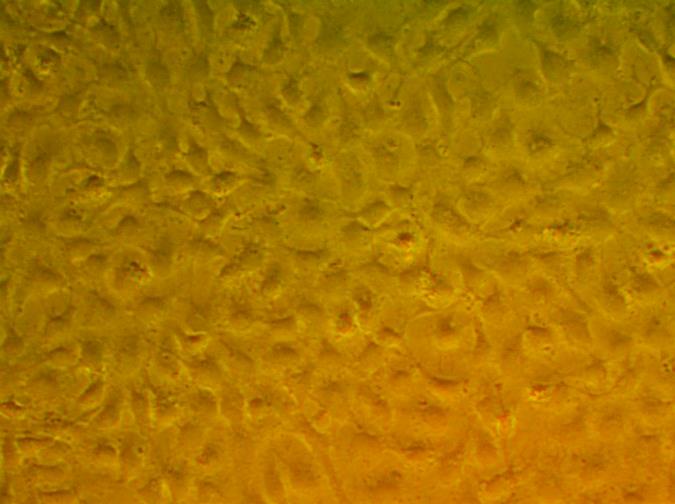

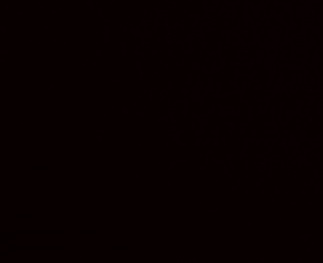

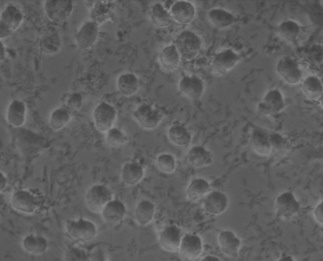

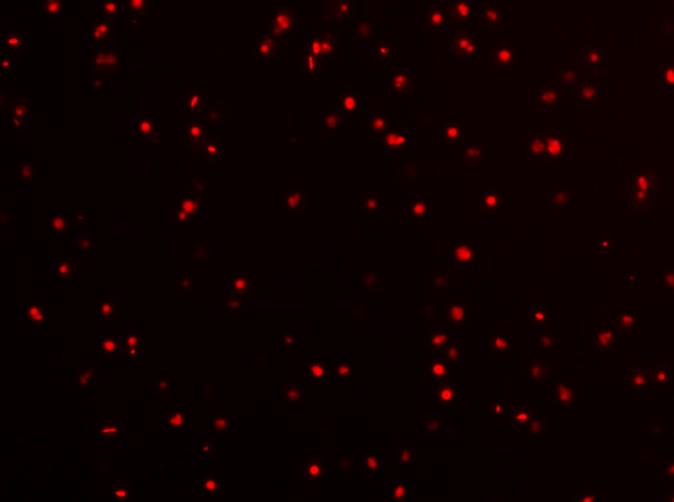

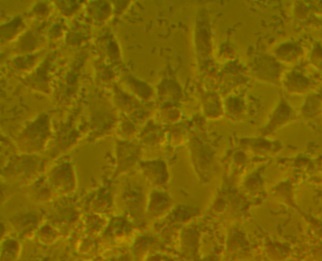

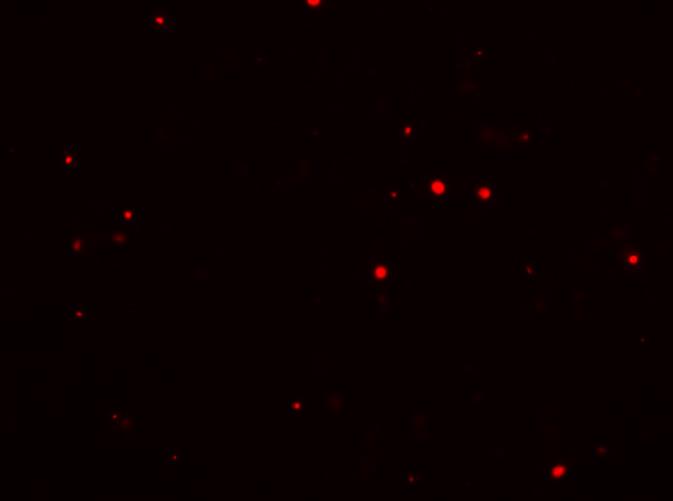

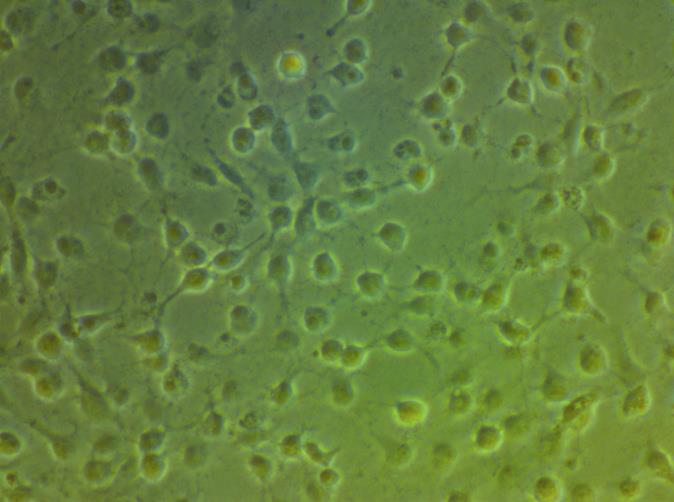

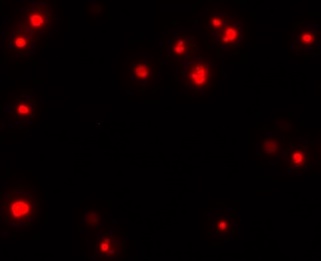

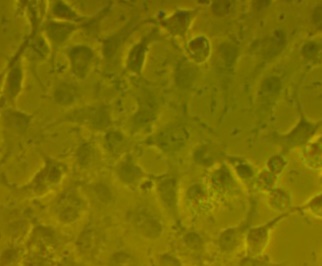

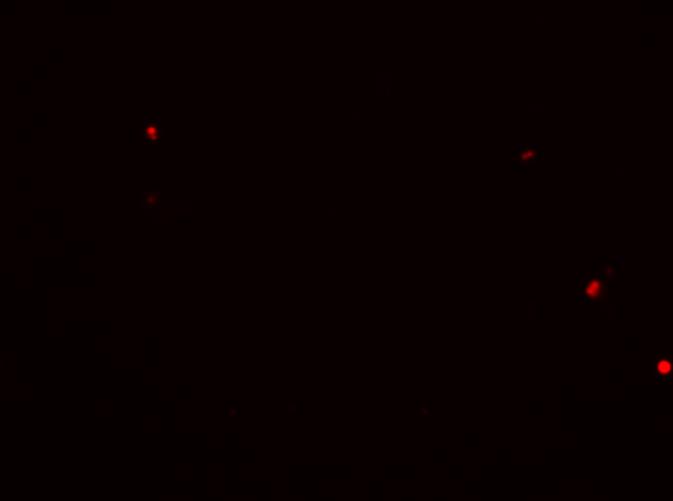

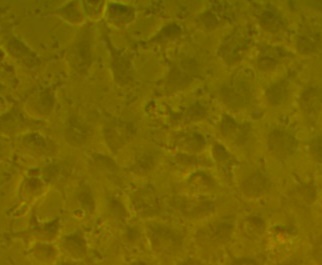

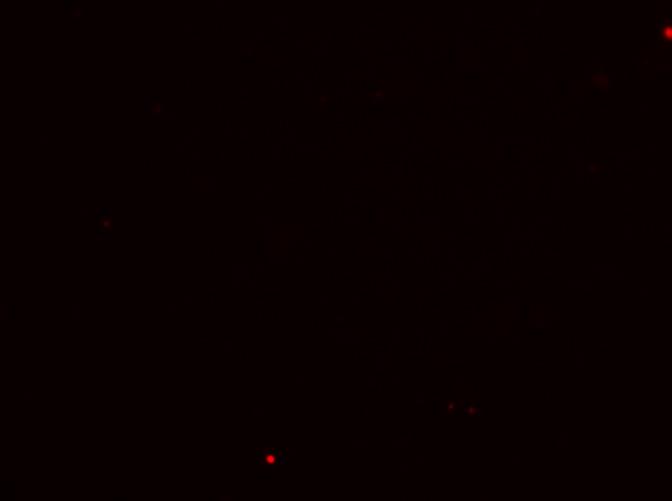

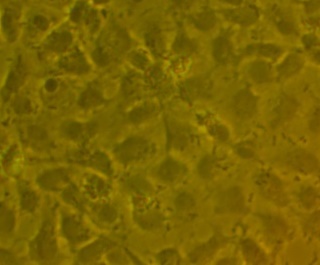

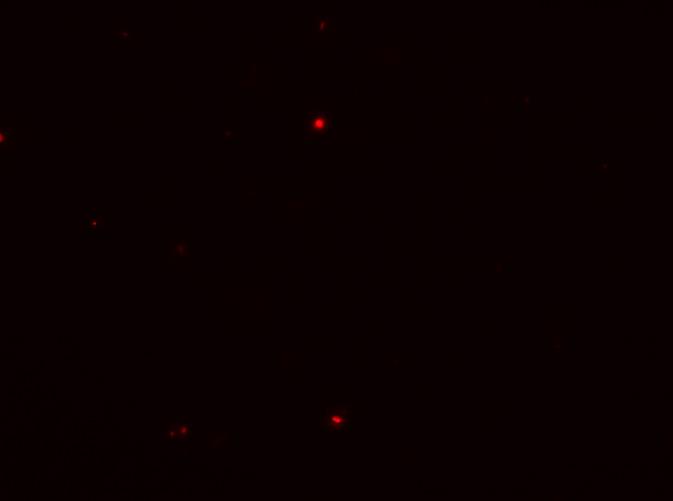


ZJO

Δ*val*1686:

p*val*1686Δ30

Δ*val*1686:

p*val*1686ΔFic

Δ*val*1686:

p*val*1686 H348A

Ctrl

Δ*val*1686

Δ*val*1686:

p*val*1686

**Figure S6 Inhibitory effect of three caspase inhibitors on *V. alginolyticus*-induced DNA fragmentation.** FHM cells were pre-incubated for 1 h with 20 μM of caspase-8 inhibitor (Q-IETD-Oph), caspase-9 inhibitor (Q-LEHD-Oph) and negative control inhibitor (Q-VE-Oph) and then infected with wild-type ZJO. After 2 h of infection, nuclear condensation and fragmentation were visualized under fluorescence microscopy after staining with Hoechst33258. Arrows indicate the fragmented nucleus. Scale bar = 50 μm.


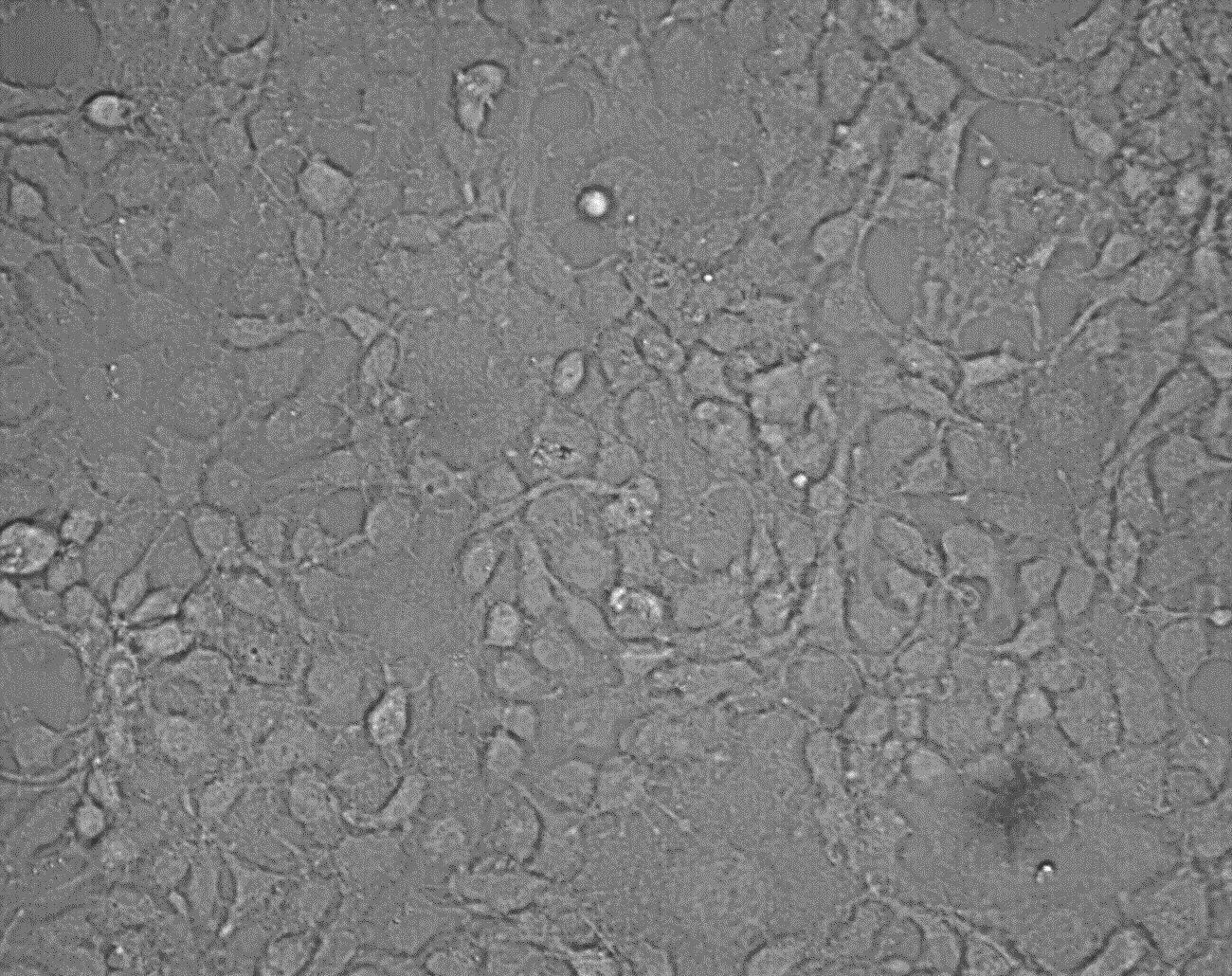

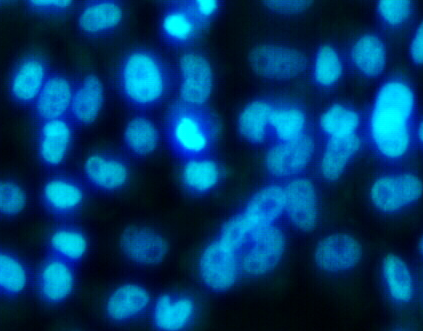


Ctrl+DMSO


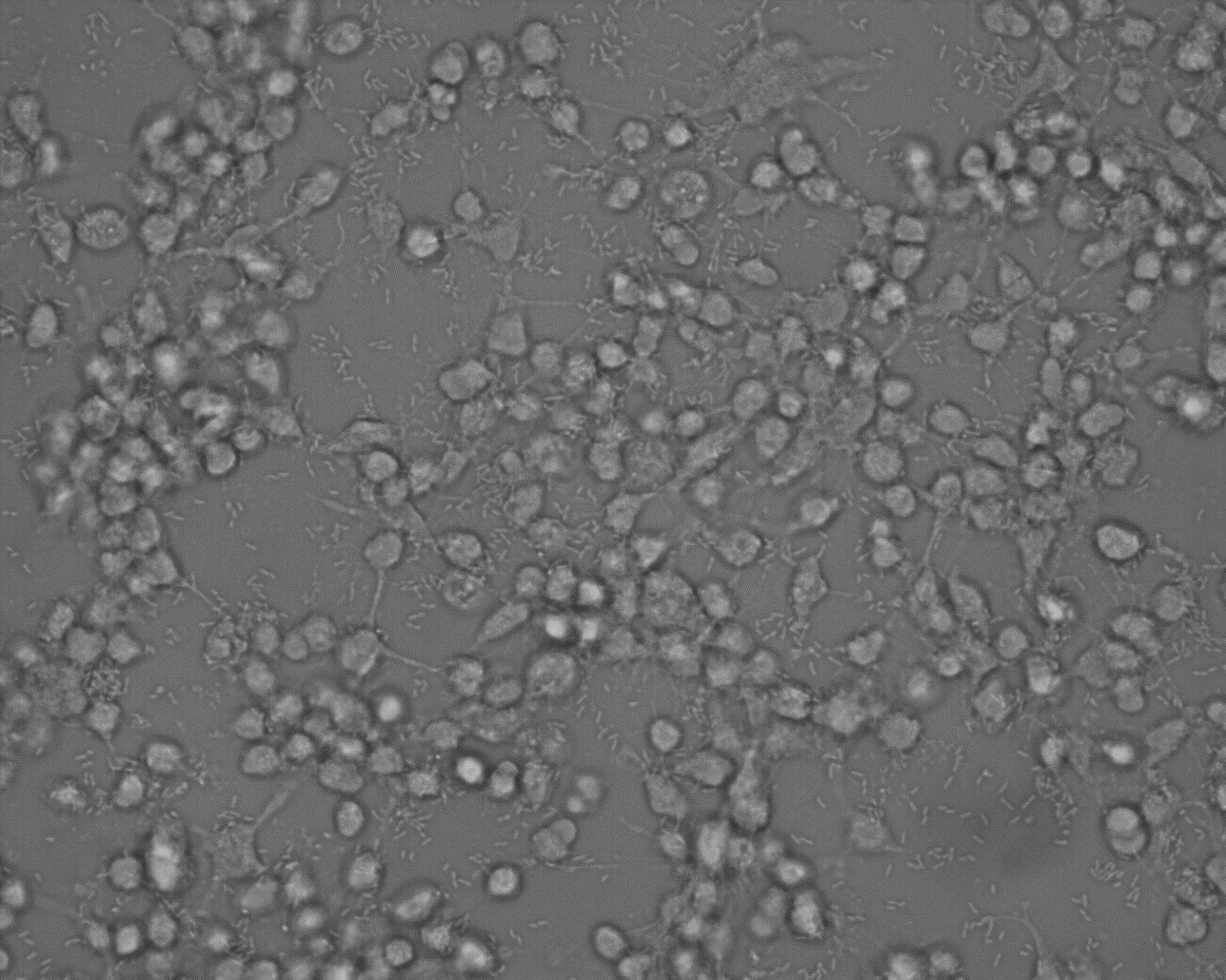

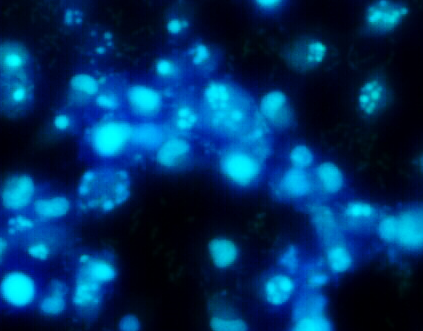


ZJO+DMSO


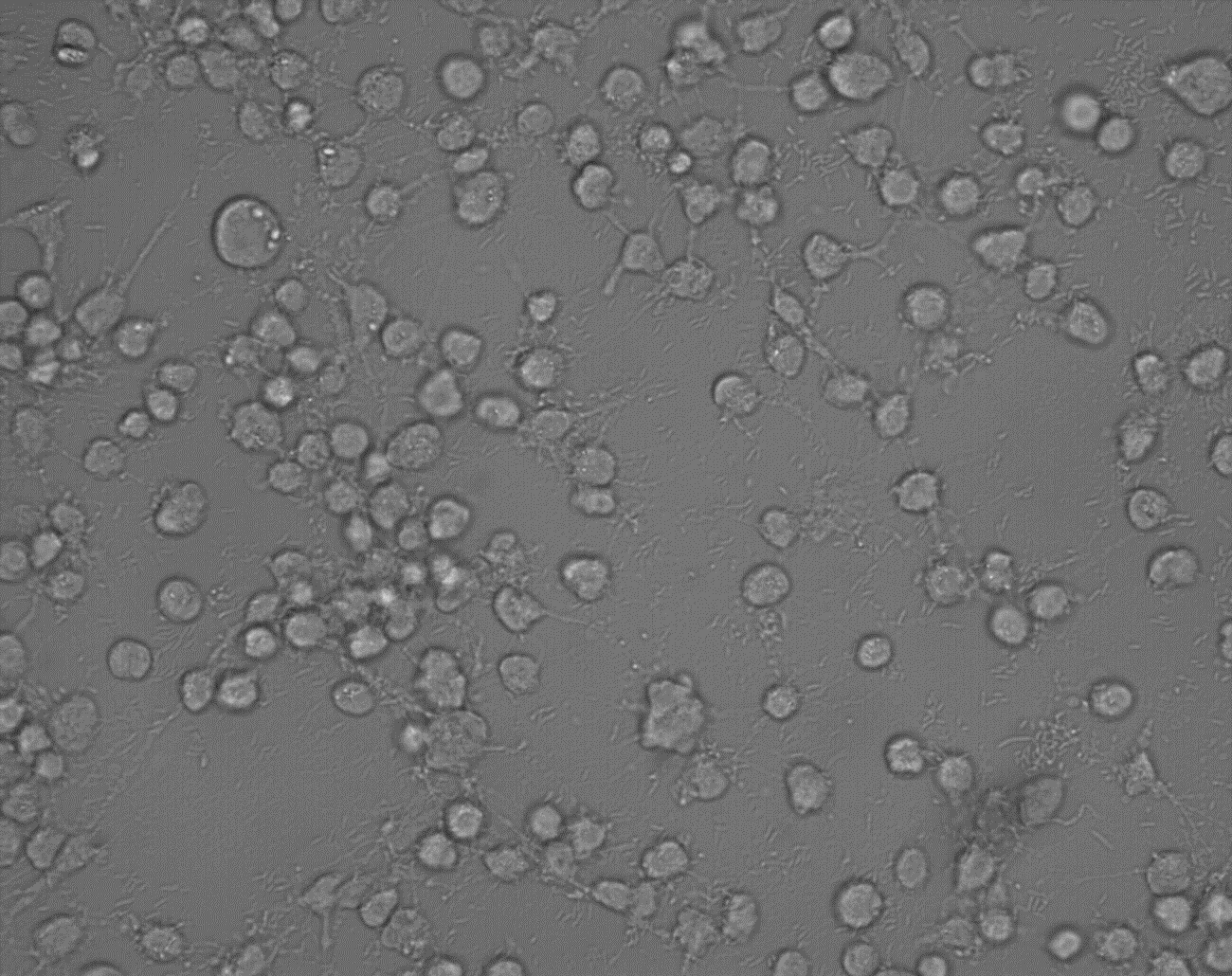

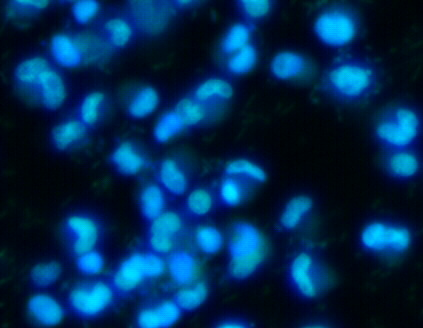


ZJO+ Q-IETD-Oph


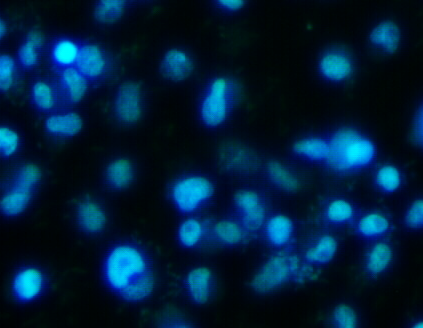

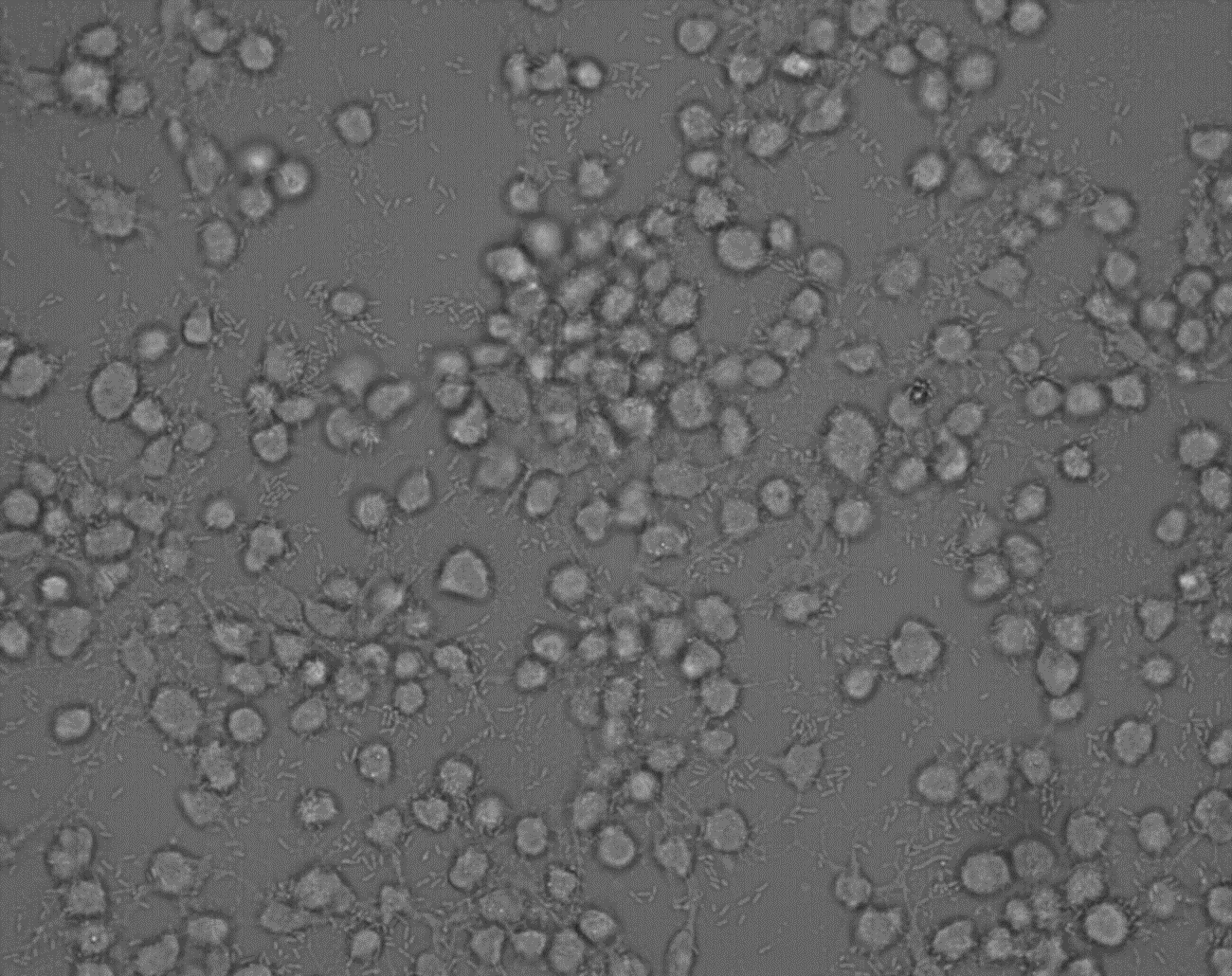


ZJO+ Q-LETD-Oph


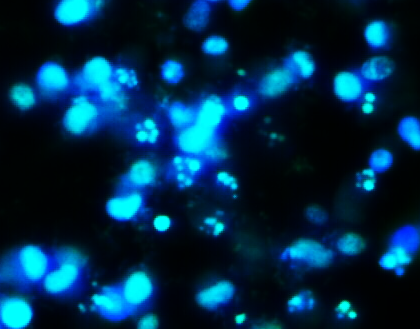

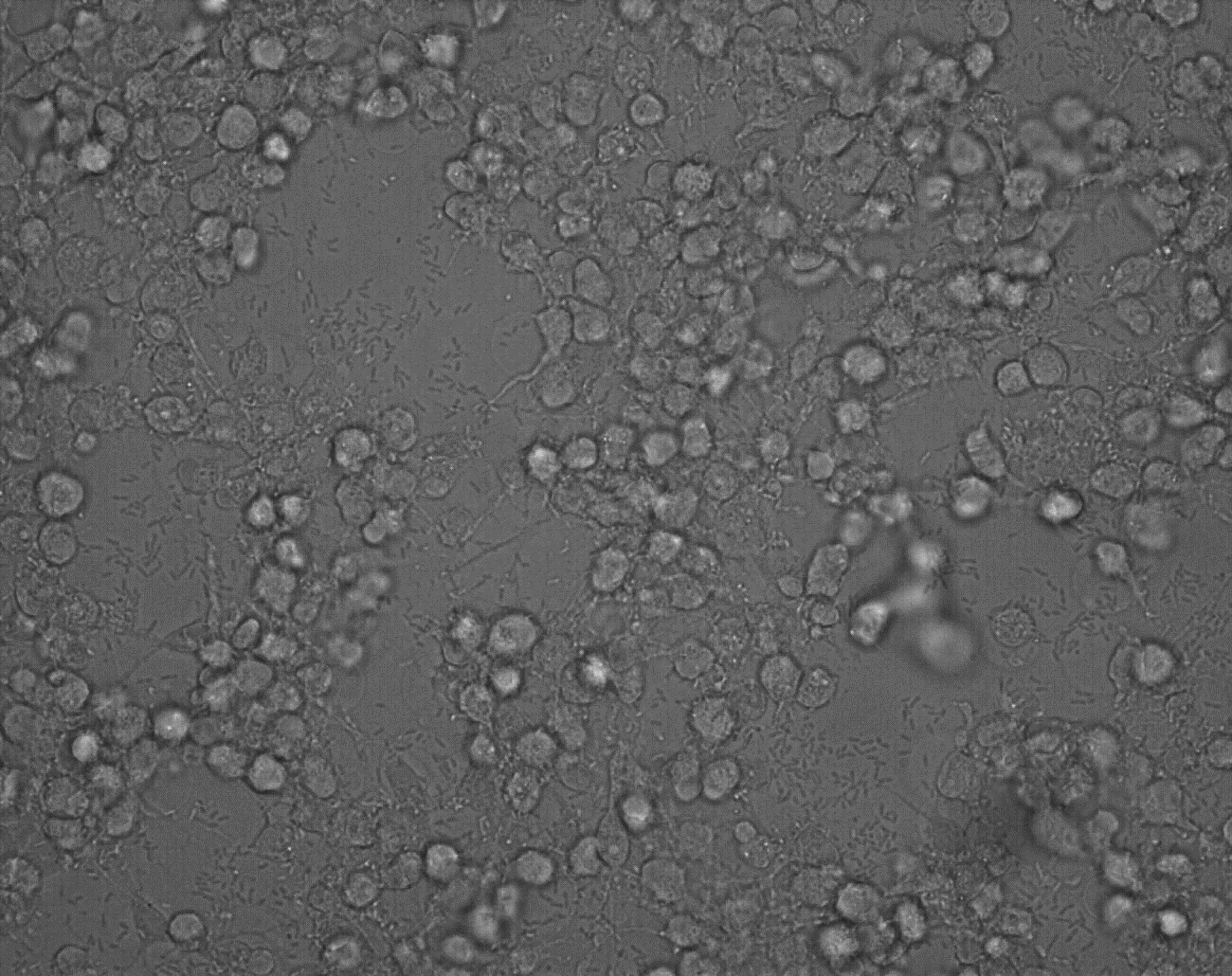


ZJO+ Q-VE-Oph

**Figure S7 Val1680 contributes to *V. alginolyticus* T3SS-induced cell lysis.**

(A) LDH release assay. FHM cells were either uninfected or infected with *V. alginolyticus* strains ZJO, Δ*val1686*, Δ*val1680*, Δ*val1686*Δ*val1680* and their corresponding complementation strains. After 2 h of infection, culture supernatants of each were measured for the release of LDH and calculated as a percentage of total cellular lysis. The data are expressed as means ± SEM from three independent experiments. The data indicates that the phenotype of LDH release was restored after complementation of the Δ*val1680* in Δ*val1680* knockout strains. (B) Microscopic observation of Δ*val*1686 Δ*val*1680 and Δ*vsc*C-infected cells after 5 h of infection. Scale bar = 10 μm.

(A)

(B)

Δ*vscC*

Phase


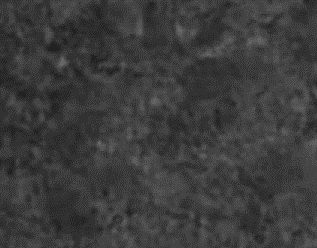

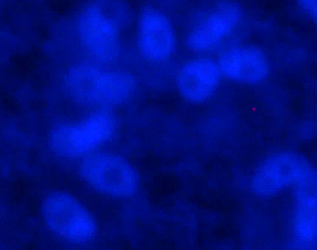

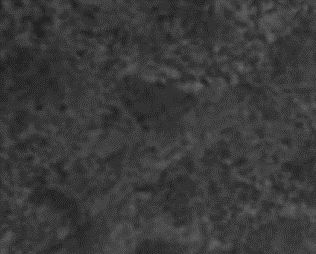

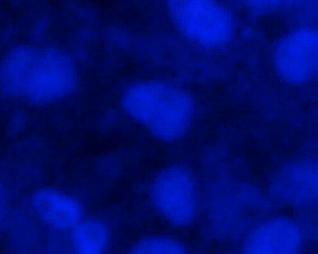


Δ*val*1686 Δ*val*1680

Hoechst
