# Supplementary material for: The Vibrio alginolyticus T3SS effectors, Val1686 and Val1680, induce cell rounding, apoptosis and lysis of fish epithelial cells
Source: Virulence. 2018 Feb 27;9(1):318–30. doi: 10.1080/21505594.2017.1414134 (PMC5955196; doi:10.1080/21505594.2017.1414134)
Supplement: KVIR_I_141314.zip [file kvir-09-01-1414134-s001.zip › KVIR_I_141314/2017VIRULENCE0242R1-s03.docx]

**Supplemental Table S1.** Strains and plasmids used in this study.

| **Strains and plasmids** | **Relevant characteristics** | **Reference** |
| --- | --- | --- |
| ***V. alginolyticus*** | | |
| ZJO | Opaque variant of wild type strain ZJ51 | Chen et al., 2009 |
| △*vscC* | *vscC* knockout which is dysfunctional in protein secretion via the T3SS apparatus | Zhao et al., 2010 |
| Δ*val1686* | *val1686* knockout | This study |
| Δ*val1680* | *val1680* knockout | This study |
| Δ*val1686*Δ*val1680* | *val168* and *val1680* double-knockout | This study |
| Δ*val1686*:p*val1686* | *val1686* knockout complemented with *val1686* | This study |
| Δ*val1686*:p*val1686*Δ30 | *val1686* knockout complemented with truncated mutant *val1686* (1-30 deletion) | This study |
| Δ*val1686*:p*val1686*ΔFic | *val1686* knockout complemented with small deletion mutant *val1686* (Fic deletion) | This study |
| Δ*val1686*:p*val1686* H348A | *val1686* knockout complemented with point mutant *val1686* (H348A) | This study |
| Δ*val1680*:p*val1680* | *val1680* knockout complemented with *val1680* | This study |
| Δ*val1686*Δ*val1680*: p*val1686* | *val168val1680* double-knockout complemented with *val1686* | This study |
| Δ*val1686*Δ*val1680*: p*val1680* | *val168val1680* double-knockout complemented with *val1680* | This study |
| ***E.coli*** | | |
| S17-1λ*pir* | *thi pro hsdR hsdM^+^ recA* RP4-2-Tc::Mu-Km::Tn7 λ*pir* | Milton *et al.* (1992) |
| S17:pDM4_∆*val1686* (A1+A2) | S17-1 strain carrying the plasmid pDM4_∆*val1686* (A1+A2) | This study |
| S17:pDM4_∆*val1680* (A1+A2) | S17-1 strain carrying the plasmid pDM4_∆*val1680* (A1+A2) | This study |
| S17:pMMB207_*val1686* | S17-1 strain carrying the plasmid pMMB207_*val1686* | This study |
| S17:pMMB207_*val1686*Δ30 | S17-1 strain carrying the plasmid pMMB207_*val1686*Δ30 | This study |
| S17:pMMB207_*val1686*ΔFic | S17-1 strain carrying the plasmid pMMB207_*val1686*ΔFic | This study |
| S17:pMMB207_*val1686*H348A | S17-1 strain carrying the plasmid pMMB207_*val1686* H348A | This study |
| S17:pMMB207_*val1680* | S17-1 strain carrying the plasmid pMMB207_*val1680* | This study |
| **Plasmids** | | |
| pDM4 vector | Cm^r^, suicide vector with an R6K origin (pir-requiring) and *sac*BR of *Bacillus subtilis* | Milton et al.1996 |
| pMMB207 vector | Cm^r^, RSF1010 derivative, *IncQ* *lacI*^q^ Tac *ori*T | Morales et al. 1991 |
| pDM4_∆*val1686* (A1+A2) | pDM4 containing the flanking region sequences of *val1686* | This study |
| pDM4_∆*val1680* (A1+A2) | pDM4 containing the flanking region sequences of *val1680* | This study |
| pMMB207_*val1686* | pMMB207 containing the *val1686* with a His.tag at the C-terminus | This study |
| pMMB207_*val1686*Δ30 | pMMB207 containing the truncated mutant *val1686* (1-30 deletion) with a His.tag at the C-terminus | This study |
| pMMB207_*val1686*ΔFic | pMMB207 containing the small deletion mutant *val1686* (Fic deletion) with a His.tag at the C-terminus | This study |
| pMMB207_*val1686*H348A | pMMB207 containing the point mutant *val1686* (H348A) with a His.tag at the C-terminus | This study |
| pMMB207_*val1680* | pMMB207 containing the *val1680* with a His.tag at the C-terminus | This study |
| pEGFP-N3 vector | Kan^r^, eukaryotic expression vector which can produce a fusion protein with GFP | Clontech |
| pEGFP_*val1686* | pEGFP-N3 containing the *val1686* | This study |
| pEGFP_*val1686*Δ30 | pEGFP-N3 containing the truncated mutant *val1686* (1-30 deletion) | This study |
| pEGFP_*val1686* H348A | pEGFP-N3 containing the point mutant *val1686* (H348A) | This study |
| pcDNA3.1(+) vector | Amp^r^, eukaryotic expression vector without any tags | Invitrogen |
| pcDNA_*val1686* | pcDNA3.1 containing the *val1686* | This study |
| pcDNA_*val1686*Δ30 | pcDNA3.1 containing the truncated mutant *val1686* (1-30 deletion) | This study |
| pcDNA_*val1686* H348A | pcDNA3.1 containing the point mutant *val1686* (H348A) | This study |

**Supplemental Table S2.** **PCR primers used in this study**

| **Primer name** | **Sequence(5’_3’)** | **Usage** |
| --- | --- | --- |
| val1686_A1_Sal I  val1686_A1_R  val1686_A2_F  val1686_A2_Spe I | ATGTCGACAAGGCTCTCCAGCGG  AAGGCTATTTTCTGCTATCTCGTTACGAGC  GCTCGTAACGAGATAGCAGAAAATAGCCTT  GCACTAGTCTTGGGTATAACGGC | construct suicide plasmid pDM4_∆val1686 (A1+A2) for val1686 knockout |
| val1686_int_F  val1686_int_R | CCACAATCGGTAGCGAAAGC  CCCTCGGTAATCACGCTCAA | confirm val1686 knockout |
| val1680_A1_Sac I  val1680_A1_R  val1680_A2_F  val1680_A2_Xho I | GTCAGAGCTCTATTACTCTGGCAACTCGTT  CCCAGCCTTCCATTTTTCTCTCCTACACAC  GAGAAAAATGGAAGGCTGGGTGTAATTTCA  GACTCTCGAGTCATTGGCATTACCTTGTTG | construct suicide plasmid pDM4_∆val1680 (A1+A2) for val1680 knockout |
| val1680_int_F  val1680_int_R | AACCTTCCACATTCCACAA  CCTTCAGCCGACAGTATC | confirm val1680 knockout |
| val1686_207_EcoR I  val1686_207_Sal I | CCGGAATTCATGATCAGTTTTGGAAGTGTTA  ACGCGTCGACTCAATGGTGATGGTGATGATGCTTAATACCGTGAAGGCTA | construct recombinant plasmid pMMB207_*val1686* for *val1686* expression under tac promoter control |
| val1686∆30_207_ EcoR I  val1686∆30_207_Sal I | CCGGAATTCATGCAGGCTGCCATGCCGCAAG  ACGCGTCGACTCAATGGTGATGGTGATGATGCTTAATACCGTGAAGGCTA | construct recombinant plasmid pMMB207_*val1686*∆30 for expressing the truncated Val1686 (1-30 deletion) under tac promoter control |
| val1686_H348A_F  val1686_H348A_R | TTATCGGGTAcgctGGCTTTACCGATG  CTCCGGCGAACAAATGTTTAC | generate the replacement of H348 by alanine in the pMMB207_*val1686*, pEGFP_*val1686*, pcDNA_*val1686* |
| val1686_∆Fic_R  val1686_∆Fic_F | AATGGTCGCATGGGGCGT  GTACCCGATAACTCCGGC | generate the deletion of Fic(HGFTDG) in the pMMB207_*val1686* |
| val1680_207_EcoR I  val1680_207_Sal I | CCGGAATTCATGGTTAATACAACACAA  ACGCGTCGACTTAATGGTGATGGTGATGATGCACCCAGCCTTCTGCCAA | construct recombinant plasmid pMMB207_*val1680* for *val1680* expression under tac promoter control |
| val1686_N3_Xho I  val1686_N3_BamH I | AGATCTCGAGCCACCATGATCAGTTTTGGAAGTGTTA  GCTCGGATCCCTTAATACCGTGAAGGCTA | construct recombinant plasmid pEGFP_*val1686* for expressing Val1686-GFP fusion protein in fish cells |
| val1686_pc3.1_BamH I  val1686_pc3.1_Xho I | GCTCGGATCCAACATGATCAGTTTTGGAAGTGT  AGATCTCGAGTCACTTAATACCGTGAAGGCTA | construct recombinant plasmid pcDNA_*val1686* for expressing Val1686 protein in fish cells |
| val1686Δ30_N3_ Xho I  val1686Δ30_N3_BamH I | AGATCTCGAGCCACCATGGGCAAAGAATACCAAATTAACGCA  GCTCGGATCCCTTAATACCGTGAAGGCTA | construct recombinant plasmid pEGFP_*val1686*Δ30 for expressing Val1686Δ30-GFP fusion protein (1-30 deletion) in fish cells |
| val1686Δ30_pc3.1_ BamH I  val1686Δ30_pc3.1_Xho I | GCTCGGATCCAACATGGGCAAAGAATACCAAATTAACGCA  AGATCTCGAGTCACTTAATACCGTGAAGGCTA | construct recombinant plasmid pcDNA_*val1686*Δ30 for expressing the truncated Val1686 (1-30 deletion) in fish cells |
